# Zoonotic *Babesia*: A scoping review of the global evidence

**DOI:** 10.1371/journal.pone.0226781

**Published:** 2019-12-30

**Authors:** Kaitlin M. Young, Tricia Corrin, Barbara Wilhelm, Carl Uhland, Judy Greig, Mariola Mascarenhas, Lisa A. Waddell

**Affiliations:** 1 Public Health Risk Sciences Division, National Microbiology Laboratory, Public Health Agency of Canada, Guelph, Ontario, Canada; 2 Big Sky Health Analytics, Vermilion, Alberta, Canada; 3 Independent Consultant, St-Hyacinthe, Quebec, Canada; University of North Dakota School of Medicine and Health Sciences, UNITED STATES

## Abstract

**Background:**

Babesiosis is a parasitic vector-borne disease of increasing public health importance. Since the first human case was reported in 1957, zoonotic species have been reported on nearly every continent. Zoonotic *Babesia* is vectored by *Ixodes* ticks and is commonly transmitted in North America by *Ixodes scapularis*, the tick species responsible for transmitting the pathogens that also cause Lyme disease, Powassan virus, and anaplasmosis in humans. Predicted climate change is expected to impact the spread of vectors, which is likely to affect the distribution of vector-borne diseases including human babesiosis.

**Methods:**

A scoping review has been executed to characterize the global evidence on zoonotic babesiosis. Articles were compiled through a comprehensive search of relevant bibliographic databases and targeted government websites. Two reviewers screened titles and abstracts for relevance and characterized full-text articles using a relevance screening and data characterization tool developed *a priori*.

**Results:**

This review included 1394 articles relevant to human babesiosis and/or zoonotic *Babesia* species. The main zoonotic species were *B*. *microti*, *B*. *divergens*, *B*. *duncani* and *B*. *venatorum*. Articles described a variety of study designs used to study babesiosis in humans and/or zoonotic *Babesia* species in vectors, animal hosts, and *in vitro* cell cultures. Topics of study included: pathogenesis (680 articles), epidemiology (480), parasite characterization (243), diagnostic test accuracy (98), mitigation (94), treatment (65), transmission (54), surveillance (29), economic analysis (7), and societal knowledge (1). No articles reported predictive models investigating the impact of climate change on *Babesia* species.

**Conclusion:**

Knowledge gaps in the current evidence include research on the economic burden associated with babesiosis, societal knowledge studies, surveillance of *Babesia* species in vectors and animal hosts, and predictive models on the impact of climate change. The scoping review results describe the current knowledge and knowledge gaps on zoonotic *Babesia* which can be used to inform future policy and decision making.

## Introduction

Babesiosis is a vector-borne disease caused by infection with erythrocytic protozoal parasites of the genus *Babesia*. There are over 100 species of *Babesia*, many of which are well-established threats to domestic animals and livestock [1, 2]. Only a few species are known to be capable of causing infection in humans [3]. Since the first case of human babesiosis was reported in 1957 in a Croatian splenectomised farmer [4], zoonotic *Babesia* species have been reported on nearly every continent. In North America, *B*. *microti* is the most reported cause of infection, with the majority of cases originating from the Northeast and upper Midwest United States [5]. In Europe, *B*. *divergens* remains the most frequent cause of human babesiosis [6]. Other zoonotic species that have been reported in the literature include *B*. *duncani* and *B*. *venatorum*, in addition to other related and unnamed *Babesia*.

Zoonotic *Babesia* species are vectored by ticks of the genus *Ixodes* [2] and require a mammalian reservoir host, such as the white-footed mouse, to complete their life cycle. The presence of different *Babesia* species around the world is dependent on the geographic distribution of competent vectors. The most common vectors of zoonotic *Babesia* species are *Ixodes scapularis*, *I*. *ricinus*, and *I*. *persulcatus* ticks in North America, Europe, and Asia, respectively [7]. Transmission to humans can occur from the bite of an infected tick [8], through the passage of contaminated blood via transfusions [9–12], or transplacentally from infected mother to fetus [13]. Typically, infection is asymptomatic and self-limiting in healthy persons. Common mild symptoms include flu-like manifestations such as fever, chills, headache, fatigue, loss of appetite, nausea, and shortness of breath. Elderly, splenectomised, and other immunocompromised individuals are at a higher risk of severe symptomology and may experience hemolytic anemia, splenomegaly, hepatomegaly, renal failure, and even death. Infection is usually diagnosed through microscopic examination of blood smears with *Babesia* species characterized largely by their size and appearance. It is recommended that patients with mild to moderate babesiosis are treated with combination therapy of atovaquone and azithromycin and severe cases with clindamycin and quinine [14, 15]. As no vaccine currently exists, tick avoidance through personal protective measures such as repellants, protective clothing, and tick checks are recommended, especially in areas of tick endemicity [2].

Evidence suggests that *Ixodes* tick ranges are changing in part due to climate change. As an example, in Canada the *I*. *scapularis* range has been expanding and cases of babesiosis, Lyme disease, Powassan virus, and anaplasmosis are increasingly being reported in affected areas [16].

In response to the potential for expansion in Canada, a scoping review (ScR) was conducted to capture the global evidence on babesiosis and *Babesia* species that infect humans. The aim was to identify and characterize the existing research on this topic, create a repository of the published literature, and identify any knowledge gaps that may facilitate decision-making on this public health issue in North America. Several literature reviews on zoonotic *Babesia* have been published; however, to the best of our knowledge, this is the only ScR that utilizes a systematic and reproducible methodology to present an updated overview of the zoonotic *Babesia* literature.

## Methods

### Review protocol and expertise

The ScR protocol was created *a priori* to ensure reproducible, transparent, and consistent results. The protocol, which includes a list of definitions, search algorithms, abstract screening form, and data characterization and utility form, can be found in the supplementary information (S1 Protocol). The repository of relevant articles and resulting dataset is also available (S1 Dataset).

Creation of the protocol and execution of the review was completed by a team of individuals with multi-disciplinary expertise in epidemiology, public health, knowledge synthesis, risk assessment, vector-borne diseases, and information science.

### Review question and scope

The objective of this ScR was to identify relevant evidence that addresses the broad research question: What are the characteristics of the global evidence on human babesiosis and zoonotic *Babesia* species?

### Search strategy

A comprehensive search strategy using the algorithm (babesiosis and (human or humans or man or woman or people)) or (Babesia), was executed in the following bibliographic databases on February 20, 2017 and updated May 10, 2018: PubMed, Scopus, Cochrane Library, and Global Health/EMBASE. There was no constraint on publication date for this search.

To ensure an exhaustive repository of relevant articles, the capacity of the electronic search was verified by hand searching the reference lists of ten literature reviews focused or partially focused on babesiosis in humans [2, 17–25]. From this exercise, six peer-reviewed primary literature articles were identified and added to the ScR for evaluation and characterization.

An extensive complementary search for grey literature, defined in this review as materials and research produced by organizations outside of the traditional commercial or academic publishing and distribution channels (e.g. government reports of surveillance data), was conducted in Google using the algorithms employed in the electronic search. A ‘snowball’ search technique was utilized in examining the first 100 hits. Using the same search terms and algorithms as described above, targeted government country and provincial/state websites were searched. For Canada and the United States, states/provinces from which the review captured reports of human babesiosis cases were then the subject of online searches for notification/monitoring systems. European, Asian, and South American countries captured by the initial electronic database search were investigated through Google using the algorithm (Country) AND (Babesia), as well as searching within the regional Centers for Disease Control and health (e.g. Pan-American Health Organization) websites. Finally, the specialized electronic bibliographic databases OpenGrey [26] and DataCite [27] were searched for relevant grey literature. Ultimately, 22 additional references were added to the project from the grey literature search.

### Relevance screening and inclusion criteria

A relevance screening form was developed *a priori* to encompass the inclusion and exclusion criteria of the ScR. Abstracts, titles, and keywords were screened for relevance using the following question: Does this citation describe primary research on babesiosis in humans or a *Babesia* species that is pathogenic to humans (in vectors, hosts, humans, or a parasite *in-vitro* study) or a predictive model examining the impacts of climate change on *Babesia*/babesiosis? Due to limited translation resources, articles published in languages other than English or French were excluded.

### Study characterization and extraction

Full-text articles of potentially relevant citations were reviewed using the data characterization and utility form. This form was developed *a priori* and designed to confirm article relevance, study design, population, outcomes, data utility, and capture whether the article addressed the impacts of climate change on the parasite.

### Scoping review management, data charting, and analysis

Citations found by the search strategy were imported into the reference management software, EndNote (EndNote X7, Clarivate Analytics), where duplicates were removed. These citations were then imported into the online electronic systematic review management platform DistillerSR (DistillerSR, Evidence Partners, Ottawa, Canada), where all stages of the ScR, including relevance screening and data characterization, were completed independently by two reviewers. Any conflicts that arose were mediated by a third reviewer.

Data collected from the data characterization and utility form were exported to Excel (Excel 2010, Microsoft Corporation, Redmond, WA), for formatting and descriptive analysis (frequencies and percentages). This process facilitated categorization and charting of relevant findings.

## Results

### Descriptive statistics

**Fig 1:**
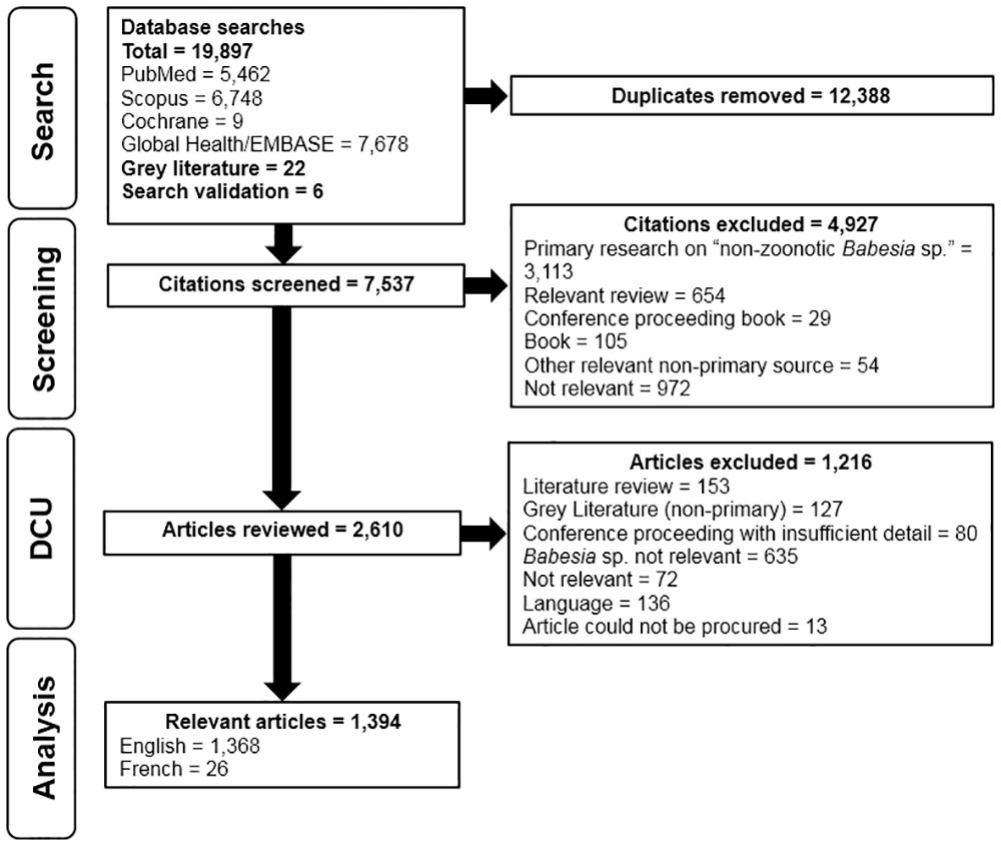
PRISMA flow diagram for scoping review of babesiosis and zoonotic *Babesia* literature.

After an extensive literature search and de-duplication process, 7537 abstracts and titles were screened for relevance from which 2610 full-text articles were reviewed. A total of 1394 relevant research articles (English = 1368 and French = 26) on zoonotic *Babesia* were characterized in this ScR (Fig 1 and S1 Dataset). Exclusions due to language (n = 136 articles) included: Chinese (n = 23), Czech (3), Danish (n = 2), Dutch (n = 5), German (n = 45), Hebrew (n = 1), Hungarian (n = 1), Italian (n = 3), Japanese (n = 5), Polish (n = 6), Portuguese (n = 2), Russian (n = 12), Serbo-Croatian (n = 1), Spanish (n = 20), Swedish (n = 1), and Turkish (n = 6) (S1 Exclusions). The major foci of these excluded articles were pathogenesis (n = 35), epidemiology (n = 45), *in vitro Babesia* studies (n = 13), transmission (n = 6), mitigation (n = 5), treatment (n = 4), and diagnostic test accuracy (n = 4). We were unable to identify a study focus for 29 excluded articles due to limited information in English or French. Relevant articles were published between 1957 and 2018, with 65% (901/1394) published since the year 2000. The majority of these articles were peer-reviewed research (1304/1394) and most frequently covered topics on pathogenesis (signs, symptoms, and diagnosis) (680/1394), and epidemiology (480/1394). Articles utilized observational (845/1394) or experimental (532/1394) study designs to investigate zoonotic *Babesia* (Table 1). Individual articles discussed throughout this ScR could have reported the results of one or more studies. Conference proceedings (n = 65), grey literature (n = 19), theses (n = 4), and predictive models (n = 2) containing primary data made up a small percentage of the articles included.

**Table 1:** General characteristics of 1394 primary research publications on babesiosis and zoonotic *Babesia*.

Category ^a^	Count
Type of Document	
Primary peer-reviewed research	1304
Conference proceeding	65
Grey literature with primary data	19
Thesis	4
Predictive Model	2
Continent	
North America	615
Europe	547
Asia	177
Africa	33
Australasia	21
Central America/South America/Caribbean	9
Zoonotic *Babesia* species	
*B*. *microti*	902
*B*. *divergens*	344
*B*. *venatorum*	101
*B*. *duncani*	35
*B*. *microti-like*	21
*B*. *divergens-like*	17
*B*. *crassa-like*	1
Unnamed	11
Not specified ^b^	4
Population Category	
Humans	534
Animal models	375
Ticks	266
Animal hosts	215
Parasite only	179
Study Design	
Observational	845
Case series/case report	387
Prevalence survey	236
Cross-sectional	201
Surveillance/monitoring	28
Cohort	17
Longitudinal	12
Case-control	6
Outbreak investigation	2
Population based case series	1
Other ^c^	1
Experimental	532
Challenge trial	425
Molecular characterization	150
Controlled trial	24
Quasi experiment	3
Evaluation of diagnostic tests	95
Economic model	4
Molecular epidemiology	2
Risk assessment	1

^a^ Category totals do not necessarily equal 1394 as articles could have reported on multiple countries, species, populations, and study designs.

^b^ Only serology conducted on human samples.

^c^ A survey of testing practices and volume of babesiosis in commercial laboratories.

**Fig 2:**
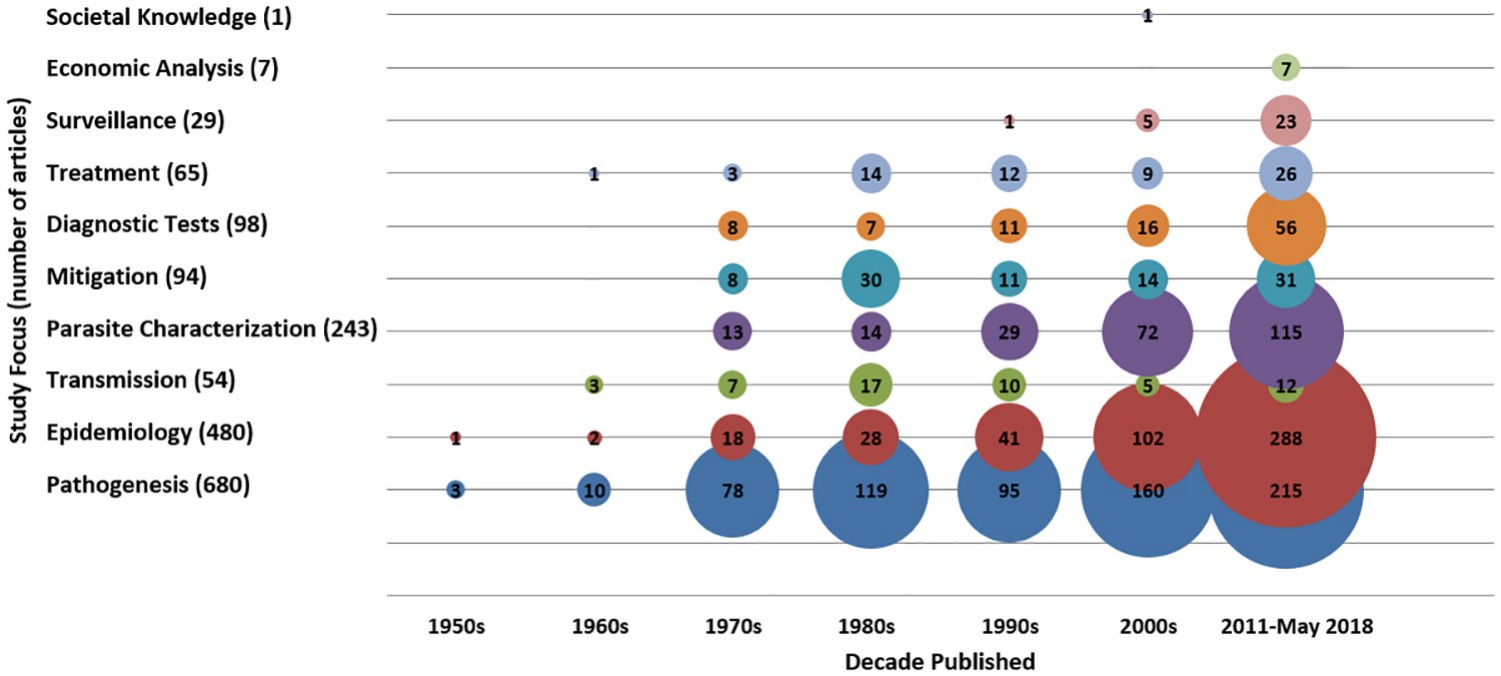
Bubble chart of the 10 major babesiosis and *Babesia* focus areas by year of publication. Bubble size is proportional to number of articles. Articles could have had more than one study focus.

Other foci of study included *in vitro*, *in silico*, and molecular morphology of zoonotic *Babesia* parasites (i.e. parasite characterization) (243/1394), articles evaluating mitigation strategies or interventions (94/1394), evaluation of diagnostic tests (98/1394), investigation of treatments (65/1394), studies on transmission or parasite competence (54/1394), description of *Babesia* surveillance activities (29/1394), economic burden or cost-benefit analysis (7/1394), and social impacts and knowledge (1/1394) (Fig 2). There were no articles captured on predictive models investigating the impact of climate change on *Babesia* species. All definitions of study characteristics, categories, and outcomes can be found in the included ScR protocol (S1 Protocol).

Humans were sampled in 534 articles (534/1394), of which 368 were case series or case reports (Table 1). Cattle were the most frequently sampled animal hosts (59/215) followed by 33 articles on bank voles, a common host of *Ixodes* species in Europe [28] and 22 articles on white-footed mice, which are cited as a common reservoir species for *Babesia* in North America [2]. There were 375/1394 articles that reported studies investigating *Babesia* using animal model species (Table 2), while 179/1394 reported the examination of parasites using cell culture methods. Ticks were the only vectors investigated (266/1394).

**Table 2:** The number of articles utilizing experimental animal models to investigate babesiosis across different study foci (N = 375).

Experimental Animal Model Category^b^	Study Focus^a^				
	Pathogenesis	Treatment	Diagnostic test accuracy	Transmission	Mitigation
Mice (n = 201)	147	27	14	11	37
Cattle (n = 6)	45	4	13	5	20
Hamsters (n = 58)	39	7	3	14	7
Gerbils (n = 48)	27	7	4	8	15
Rats (n = 14)	13	-	-	1	3
Voles (n = 11)	6	-	-	4	-
Monkeys ^c^ (n = 11)	11	-	-	2	-
Sheep (n = 7)	7	-	-	1	1
Dogs (n = 5)	4	-	1	-	-
Deer (n = 3)	3	-	-	1	-
Rabbits (n = 2)	-	-	-	2	-
Reindeer (n = 1)	1	-	-	1	-
Elk (n = 1)	1	-	-	-	-
Other ^d^ (n = 3)	2	-	1	-	-

^a^ Study focus total may not add to animal model category as a single study may contain results for more than one focus area.

^b^ Animal model category totals do not add up to total number of articles as a single study may contain results for more than one animal model.

^c^ including chimpanzees.

^d^ Other animal species studied included: chicks, pigs, goats, lemmings and horses.

Evidence of the capacity for human infection was identified for seven *Babesia* species (Table 1). These included *B*. *microti*, *B*. *divergens*, *B*. *duncani*, *B*. *venatorum*, *B*. *microti-like*, *B*. *divergens-like*, and *B*. *crassa-like*. There are also several variants that have been associated with human infection but remain unnamed. These include *B*. *sp*. *KO1*, *B*. *sp*. *TW1*, *B*. *sp*. *MO1*, *B*. *sp*. *CA1*, *B*. *sp*. *WA2 (*aka *B*. *duncani-type)*, and *B*. *sp*. *XXB/HangZhou*. From here, reference to *Babesia* species refers to only those known to infect humans (i.e. zoonotic).

### *Babesia* characterization

*Babesia* characterization, meaning studies of the parasite(s) with no affiliation to a host, was the focus of 243/1394 articles. Cell cultures were used to investigate parasite pathogenesis, including immune reactions and production of various antigens/antibodies (77/243), as well as to examine the transmission characteristics of the parasite that facilitate movement from one host to another (e.g. receptors and proteins) (9/243). The genetic makeup of the pathogen to determine relatedness of isolates and/or identify key areas of conservation or mutation was reported in 182/243 articles. Many of these articles reported phylogenetic trees (114/182) and/or whole genome sequencing (7/182) in their study analyses.

Molecular epidemiology, which examines the link between epidemiological information and genetics of the pathogen, was the focus of two articles (2/243) [29, 30].

### Transmission routes and scenarios

In articles that reported on human sampling frames (534/1394), tick bites were the most common reported source of exposure to *Babesia* (155/534), followed by blood transfusion (78/534). Four studies reported human exposure through direct contact with infected animals or tick infested animals [31–34]. Transplacental transmission of *B*. *microti* in humans from mother to fetus has been reported in ten studies. Two of these were surveillance reports [5, 35] and eight were case reports describing newborns with congenital infections [36–43]. None of the infants were reported to have long-term sequelae from the congenital *B*. *microti* infection. The source of exposure was not described in 349/534 articles that sampled humans.

Blood transfusion transmission scenarios were studied in two experiments. In a challenge study, Rhesus macaque monkeys were used to investigate parasitemia kinetics and immune responses following transfusion of blood infected with *B*. *microti* [44]. Another challenge study analyzed the viability of *B*. *microti* infected blood stored at room temperature or 4°C [45].

Conditions for transmission of zoonotic *Babesia* species to, from, and between humans, animals, or vectors was the focus of 54/1394 articles. Host-to-host transmission scenarios were reported in 8/54 of the transmission articles. These included observational studies that documented transplacental transmission in naturally-infected populations of white-footed mice and meadow voles in endemic areas of the United States [46], as well as common voles, tundra voles, and field voles in Poland [47]. Similarly, transplacental transmission has also been demonstrated experimentally in a population of BALB/c lab mice [48]. Other host-to-host studies described experimental attempts at the induction of infection through inoculation of infected blood from cattle to reindeer [49], cattle to sika deer [50], macaque to macaque [44], and between desert woodrats, dusty-footed woodrats, California voles, California mice, and deer mice [51]. One study, published in 1994, demonstrated oral transmission of *Babesia* through oral gavage and cannibalism in laboratory mice [52].

Host to vector (21/54) and vector to host (22/54) transmission scenarios involved the experimental feeding of ticks on infected animals, or infected ticks on *Babesia*-free animals (S1 Table). These studies examined whether sufficient parasitemia was reached in animal species to transmit *Babesia* to ticks and whether various tick species were competent vectors of zoonotic *Babesia* species. Many of these studies also monitored infection kinetics throughout the course of infection.

Only 15/54 of the transmission articles specifically reported on the competency of vectors to transmit *Babesia*. The most frequent outcome was transmission potential defined as the presence of the parasite within the salivary gland of the tick (12/15), demonstrated by *B*. *microti* in *I*. *scapularis* [8, 53–58]; *B*. *venatorum* [59], *B*. *divergens* [60], and *B*. *microti* in *I*. *ricinus* [61, 62]; and *B*. *microti* in *I*. *ovatus* and *I*. *persulcatus* [63]. Competency studies also investigated the course of *B*. *microti* metamorphosis in the gut of *I*. *scapularis* ticks [58, 64, 65] and *B*. *divergens* metamorphosis in the gut of *I*. *ricinus* ticks [60], as well as the ability of *B*. *microti* to cross the peritrophic membrane in *I*. *scapularis* ticks [66].

Vector to vector transmission (7/54) included investigations of transstadial and/or transovarial transmission of *B*. *microti* in *I*. *ricinus* (2/7), *I*. *trianguliceps* (1/7), *D*. *andersonii* (1/7) and *R*. *heamaphysaloides* (1/7) and *B*. *divergens* in *I*. *ricinus* (2/7).

### Diagnostic testing and accuracy

Many studies captured in this review utilized a form of testing for the purpose of either identifying *Babesia* in patients, animals, and ticks, screening host populations for antibodies indicative of prior infection, or for the evaluation of their accuracy in diagnosing human cases and/or detecting *Babesia* species in non-human hosts.

Blood samples were taken in 94.6% (505/534) of articles reporting human sampling frames to determine *Babesia* infection or sero-positivity in humans. Evidence of *Babesia* infection was also demonstrated in the erythrocytes of the spleen [67–70], kidney [71], lymph node, lung, and liver [69], as well as in the bone marrow [4, 72–83], cerebral spinal fluid [84–87], placenta [37], and amniotic fluid [42] of patients. Microscopic blood smear evidence of *Babesia* was the most common method of diagnosis in humans (407/534), followed by immunoassay (302/534) and molecular (235/534) techniques. In 64/534 articles, infection status was investigated using whole animal bioassays.

In articles that reported animal sampling frames (natural hosts and animal model species), diagnosis involved microscopic blood smear examinations (422/567), immunoassays (191/567), and molecular techniques (156/567). Molecular methods were most heavily relied on for the identification of *Babesia* in ticks (230/266), while 35/266 articles reported microscopic bodily fluid exams and 4/266 immunoassays.

There were 98/1394 articles that had a diagnostic test accuracy focus. These articles reported on the accuracy of one or more diagnostic/detection tests, of which 19/98 reported on microscopic examination and staining of *Babesia* parasites in blood (n = 17), tissue (n = 1), and bodily fluid (n = 1). Serological test accuracy, including indirect fluorescent antibody technique and enzyme-linked immunosorbent assay, was reported in 48/98 articles. Molecular test accuracy, most frequently concerning polymerase chain reaction, was reported in 46/98 articles. Notably, only 53.1% (52/98) of these articles provided extractable outcome data on the sensitivity, specificity, and/or raw data on the test accuracy.

### Pathogenesis in humans and animal hosts

Pathogenesis was the focus of 680/1394 articles. Humans were studied in 390/680 of these articles, of which 368/390 were case series or case reports. These studies investigated the course of *Babesia* infection, diagnosis, clinical characteristics, and complications. Five articles also reported on the immune response elicited by infection (5/390). Signs and symptoms of disease manifestation were reported in most of the human articles (365/390), while pathology outcomes, defined as any blood, urine, or tissue results using chemistry, clinical microbiology, hematology, or molecular pathology to characterize the progression of disease, were reported in less than half (181/390). Post-mortem findings (14/390) and chronic sequelae resulting from babesiosis infection (4/390) were rarely mentioned in the literature. The intrinsic incubation period, also known as the time between infection and the onset of symptoms, was reported in 6/390 articles.

Challenge or controlled trials were used to investigate pathogenesis in experimental animal models (266/680). In addition to describing the course of *Babesia* infection (160/266), these experiments were used to investigate the infection mechanism (19/266) and immune response (131/266) in the animal. These trials allowed investigators to study infection characteristics including signs and symptoms (68/266), the latent period (139/266), intrinsic incubation period (2/266), and infectious period (7/266) of the experimental animal model species.

Animal case reports provided results of pathology (4/20) and post-mortem examinations (3/20). Specific signs and symptoms of animals naturally infected with *Babesia* were reported in over half of the case reports (11/20). No long-term sequelae were reported in any of the animal studies.

### Coinfections

Many articles reported coinfection (209/1394) of zoonotic *Babesia* with other pathogens. Coinfections were noted in 124/534 articles that reported on humans. These included case reports (77/124), cohort studies (4/124), case-control studies (2/124), cross-sectional studies (18/124), prevalence surveys (22/124), surveillance programs (6/124), and a longitudinal study (1/124) that identified the presence of *Babesia* and other pathogens. Lyme disease, caused by *Borrelia burgdorferi*, was the most common coinfection (99/124) followed by ehrlichiosis (17/124), anaplasmosis (15/124), bartonellosis (3/124), and rickettsioses (2/124). One article (1/124) reported coinfection with two or more *Babesia* species simultaneously [88].

Of the articles that reported on animal host populations in which zoonotic *Babesia* were detected, 30/173 reported at least one coinfection as an outcome. These included the bacteria; *Borrelia* (8/30), *Anaplasma* (8/30), *Bartonella* (5/30), *Ehrlichia* (4/30), *Grahamella* (1/30), *Candidatus* (2/30), *Leptospira* (1/30), parasites; two or more species of *Babesia* (6/30), *Trypanosoma* (2/30), *Hepatozoon* (1/30), *Theileria* (6/30), and the virus; *Dobrava-Belgrade Virus* (1/30).

Of the articles that reported on tick populations in which zoonotic *Babesia* species were detected, 57/211 reported at least one coinfection as an outcome. These included coinfections with multiple species of *Borrelia* (43/57), *Anaplasma* (21/57), *Ehrlichia* (4/57), *Rickettsia* (16/57), *Theileria* (1/57), *Bartonella* (1/57), Tick-borne Encephalitis Virus (2/57), *Toxoplasma gondii* (3/57), *Coxiella burnetii* (1/57), and *Candidatus* Neoehrlichia mikurensis (3/57).

### Epidemiology

Epidemiology was the focus of 480/1394 articles. *Babesia* infection or exposure in humans was reported in 137/480 epidemiology articles (Table 3). Most of these articles (76/137) reported general population sampling frames (e.g. blood donors or random sample of people in an area), fewer reported an “at risk” population (45/137) such as forestry workers, farmers, or individuals with a history of tick bites, or cases of clinically diagnosed babesiosis (20/137) in an area (usually a state/province) in a defined time period. Cross-sectional (45/137) and prevalence surveys (58/137) were the most popular study designs used for gathering human epidemiological data. Case prevalence, identification of the number of individuals with *Babesia* infection in a sampling frame, was a reported outcome among populations in 46/137 articles. Other outcomes included seroprevalence (89/137) and incidence (31/137). Statistically significant risk factors for exposure to *Babesia* were communicated in 28/137 articles reporting on epidemiological studies of human populations. In addition to the epidemiological studies, 372/1394 articles described human sporadic cases, with hospitalizations and/or case-fatalities reported in 332/372 and 52/372 articles, respectively (Table 3).

**Table 3:** Details of human epidemiology outcomes (N = 137) and sporadic case reports (N = 372) by continent and country.

Country (Grouped by continent)	N (# studies)	*Babesia* sp. (n) ^a^	Sero-prevalence	Case prevalence	Case Incidence	Prevalence of long-term sequelae	Case-fatality proportion	Proportion hospitalized	Proportion asymptomatic	Other ^b^	Sporadic case reports^c^	Risk Factors
**North America**												
**United States**	353	*B*. *microti* (245) *B*. *divergens* (2) *B*. *duncani* (12) *B*. *venatorum* (1) *B*. *divergens-like* (1) *B*. *sp*. *MO1* (2) *B*. *sp*. *WA2* (1) Sp. not determined (103)	59	28	29	2	11	13	1	5	274	22
**Canada**	8	*B*. *microti* (6) *B*. *duncani* (1) Sp. not determined (1)	-	-	1	-	-	-	-	-	7	-
**Mexico**	1	Sp. not determined (1)	1	-	-	-	-	-	-	-	-	-
**Europe**												
**France**	21	*B*. *microti* (5) *B*. *divergens* (12) *B*. *divergens-like* (1) Sp. not determined (7)	2	-	-	-	-	-	-	-	20	1
**United Kingdom (Great Britain, Ireland, Scotland)**	11	*B*. *microti* (1) *B*. *divergens* (6) *B*. *duncani* (1) Sp. not determined (3)	-	-	-	-	-	-	-	-	11	-
**Poland**	10	*B*. *microti* (9) Sp. not determined (1)	5	5	-	-	-	-	-	-	1	1
**Spain**	9	*B*. *microti* (2) *B*. *divergens* (4) Sp. not determined (3)	-	-	-	-	-	-	-	-	9	-
**Germany**	5	*B*. *microti* (4) *B*. *divergens* (1) *B*. *venatorum* (1)	3	-	-	-	-	-	-	-	2	-
**Italy**	5	*B*. *microti* (2) *B*. *divergens* (3) *B*. *venatorum* (3) *B*. *microti-like* (1) *B*. *divergens-like* (1)	3	-	-	-	-	-	-	-	2	1
**Austria**	4	*B*. *microti* (2) *B*. *divergens* (1) *B*. *venatorum* (3)	1	-	1	-	-	-	-	1	3	1
**Switzerland**	4	*B*. *microti* (2) *B*. *divergens* (2)	1	-	-	-	-	-	-	-	3	-
**Sweden**	3	*B*. *microti* (1) *B*. *divergens* (2) *B*. *venatorum* (2)	1	-	-	-	-	-	-	-	2	-
**Netherlands**	2	*B*. *microti* (2) *B*. *divergens* (1) *B*. *venatorum* (1)	-	1	-	-	-	-	-	-	2	-
**Belgium**	1	*B*. *microti* (1) *B*. *divergens* (1) *B*. *venatorum* (1)	1	-	-	-	-	-	-	-	-	1
**Croatia**	1	*B*. *microti* (1)	1	-	-	-	-	-	-	-	-	-
**Denmark**	1	*B*. *microti* (1)	-	-	-	-	-	-	-	-	1	-
**Finland**	1	*B*. *divergens* (1)	-	-	-	-	-	-	-	-	1	-
**Iceland**	1	*B*. *microti* (1)	1	-	-	-	-	-	-	-	-	-
**Macedonia**	1	*B*. *microti* (1)	-	-	-	-	-	-	-	-	1	-
**Montenegro**	1	Sp. not determined (1)	-	-	-	-	-	-	-	-	1	-
**Norway**	1	*B*. *divergens* (1)	-	-	-	-	-	-	-	-	1	-
**Portugal**	1	*B*. *divergens* (1)	-	-	-	-	-	-	-	-	1	-
**Romania**	1	Sp. not determined (1)	1	1	-	-	-	-	-	-	-	-
**Slovenia**	1	*B*. *divergens* (1)	1	-	-	-	-	-	-	-	-	-
**Yugoslavia**	1	Sp. not determined (1)	-	-	-	-	-	-	-	-	1	-
**Asia**												
**China**	18	*B*. *microti* (11) *B*. *divergens* (1) *B*. *venatorum* (3) *B*. *sp*. *TW1* (1) *B*. *sp*. *XXB/HangZhou* (1) *B*. *crassa-like* (1) Sp. not determined (1)	4	5	-	1	-	1	-	-	12	-
**Japan**	3	*B*. *microti* (1) *B*. *microti-like* (2)	1	-	-	-	-	-	-	-	2	-
**India**	1	Sp. not determined (1)	-	-	-	-	-	-	-	-	1	-
**Mongolia**	1	*B*. *microti* (1)	1	1	-	-	-	-	-	-	-	-
**Qatar**	1	Sp. not determined (1)	-	-	-	-	-	-	-	-	1	-
**Republic of Korea**	1	*B*. *sp*. *KO1* (1)	-	1	-	-	-	-	-	-	1	-
**Russia**	1	*B*. *microti* (1)	-	1	-	-	-	-	-	-	-	-
**Turkey**	1	*B*. *divergens* (1)	-	-	-	-	-	-	-	-	1	-
**Australasia**												
**Australia**	5	*B*. *microti* (5) *B*. *duncani* (2) Sp. not determined (1)	-	1	-	-	-	-	-	-	4	-
**Africa**												
**Egypt**	3	Sp. not determined (3)	-	-	-	-	-	-	-	-	3	-
**Cote d’Ivoire**	1	Sp. not determined (1)	-	-	-	-	-	-	-	-	1	-
**Democratic Republic of the Congo**	1	*B*. *microti* (1)	-	1	-	-	-	-	-	-	-	-
**South Africa**	1	Sp. not determined (1)	-	-	-	-	-	-	-	-	1	-
**Sudan**	1	Sp. not determined (1)	-	1	-	-	-	-	-	-	-	-
**Tanzania**	1	*B*. *microti* (1)	1	-	-	-	-	-	-	-	-	-
**South America**												
**Brazil**	2	*B*. *microti* (1) Sp. not determined (1)	1	-	-	-	-	-	-	-	2	1
**Bolivia**	1	*B*. *microti* (1)	1	-	-	-	-	-	-	-	-	-
**Columbia**	1	Sp. not determined (1)	-	-	-	-	-	-	-	-	1	-

All values are counts of individual articles.

^a^ Total of categories may be greater than the total articles for each country as some investigated more than one outcome.

^b^ Other outcomes reported included: sero-reversion, estimated risk, proportion of *Babesia* positive donors with risk factors, duration of DNA detectability, and exposure to *Babesia* reported on entry to thalassemia network.

^c^ Number of articles reporting on one or more cases. Cases were classified under country of study unless country of acquisition clearly reported.

Epidemiology articles that reported zoonotic *Babesia* in animal hosts reported case prevalence (140/173), seroprevalence (46/173), and incidence (2/173) (Table 4 and S2 Table). Prevalence surveys were the most common epidemiological study captured (92/173), followed by cross-sectional (74/173), longitudinal (7/173), cohort (5/173), and surveillance or monitoring program (3/173). Nineteen articles (19/1394) reported sporadic cases of babesiosis within animal host populations (Table 4), of which 4/19 reported case fatalities.

**Table 4:** Heat chart of the number of articles reporting zoonotic *Babesia* in animal host categories organized by continent and epidemiology outcome (N = 173), outbreaks (N = 4), and sporadic cases (N = 19).

Outcome*	Animal Category									
	Rodents (94)	Sorex (17)	Bovids (53)	Cervids (25)	Monkeys (4)	Ursids (2)	Canids (11)	Felines (3)	Leporids (4)	Others^b^ (8)
**Prevalence of confirmed zoonotic *Babesia* sp. infection** a										
**North America**	**21**	**3**	**-**	**1**	**-**	**2**	**-**	**-**	**4**	**1**
**Europe**	**30**	**3**	**22**	**16**	**-**	**-**	**7**	**2**	**-**	**3**
**Asia**	**24**	**10**	**3**	**3**	**-**	**-**	**-**	**-**	**-**	**3**
**Africa**	**6**	**-**	**1**	**-**	**2**	**-**	**-**	**-**	**-**	**-**
**Sero-prevalence**										
**North America**	**8**	**-**	**-**	**-**	**-**	**-**	**-**	**-**	**2**	**-**
**Europe**	**4**	**1**	**22**	**3**	**-**	**-**	**1**	**2**	**-**	**2**
**Asia**	**3**	**2**	**-**	**-**	**-**	**-**	**-**	**-**	**-**	**-**
**Africa**	**-**	**-**	**1**	**-**	**-**	**-**	**-**	**-**	**-**	**-**
**Outbreak of zoonotic *Babesia* sp.**										
**North America**	**-**	**-**	**-**	**-**	**-**	**-**	**-**	**-**	**-**	**-**
**Europe**	**-**	**-**	**3**	**1**	**-**	**-**	**-**	**-**	**-**	**-**
**Asia**	**-**	**-**	**-**	**-**	**-**	**-**	**-**	**-**	**-**	**-**
**Africa**	**-**	**-**	**-**	**-**	**-**	**-**	**-**	**-**	**-**	**-**
**Sporadic cases of zoonotic *Babesia* sp.**										
**North America**	**-**	**-**	**-**	**-**	**1**	**-**	**-**	**-**	**-**	**-**
**Europe**	**1**	**-**	**9**	**2**	**-**	**-**	**4**	**-**	**-**	**-**
**Asia**	**-**	**-**	**-**	**-**	**1**	**-**	**-**	**-**	**-**	**-**
**Africa**	**-**	**-**	**1**	**-**	**-**	**-**	**-**	**-**	**-**	**-**

The darker the color the more articles captured.

^a^ Case prevalence is number of individuals with infection in a defined population at a specific point in time.

^b^ Other animals included raccoons, weasels, horses, eurasian badgers, european moles, short-tailed gymnures, and unspecified ungulates.

*Incidence was a reported outcome in 2 articles.

Among the epidemiology articles, 211/480 reported evidence of zoonotic *Babesia* infection in at least one tick species. Ticks were primarily collected by the use of tick dragging (149/211), which involves dragging a white cloth over vegetation to collect questing ticks. Other articles described obtaining ticks through removal from human or animal hosts (89/211) for testing. Across epidemiology articles, all life stages were collected, however nymph (148/211) and adult (161/211) life stages were more frequently sampled than larvae (67/211).

The majority of epidemiology articles reported infection in the *Ixodes* genus of ticks (Table 5). *Babesia* was also detected in the following tick populations: *Dermacentor reticulatus* [89–96], *D*. *marginatus* [97–99], *D*. *sylvarum* [100], *Haemaphysalis longicornis* [101–103], *H*. *punctata* [98, 99, 104], *H*. *concinna* [100, 105, 106], *H*. *leporispalustris* [107], *H*. *japonica* [108], *H*. *sulcata* [109], *Hyalomma marginatum* [110, 111], *Amblyomma variegatum* [112], *Argas (Carios) vespertilionis* [113], *Rhipicephalus simus* [114], *R*. *turanicus* [115], *R*. *bursa* [116], *R*. *microplus* [101], and *R*. *sanguineus* [117]. Five articles (5/211) estimated the minimum infection rate within tick species [63, 107, 118–120].

**Table 5:** Heat chart of number of articles reporting the detection of zoonotic *Babesia* in tick species organized by country (N = 211 articles).

Country (Grouped by Continent)	Total*	I. scapularis	I. ricinus	I. persulcatus	I. trianguliceps	I. hexagonus	I. ovatus	I.canisuga	Other Ixodes spp.^a^	D.reticulatus	D. marginatus	D. sylvarum	H. longicornis	H. punctata	H. concinna	Other Haemaphysalis spp.^b^	Hy. marginatum	A. variegatum ^c^	A. vespertilionis ^d^	Rhipicephalus spp.^e^	Not speciated
Total*	**211**	**44**	**126**	**22**	**5**	**4**	**2**	**2**	**6**	**8**	**3**	**1**	**3**	**3**	**3**	**3**	**2**	**1**	**1**	**5**	**2**
**North America**																					
**United States**	**43**	**42**							**2**							**1**					
**Canada**	**2**	**2**																			
**Europe**																					
**Poland**	**27**		23							6											
**Germany**	**17**		17		1	2		1													
**Italy**	**14**		12		1												1			3	1
**France**	**10**		9								1										
**Switzerland**	**10**		9		1						1			1							
**Netherlands**	**7**		7																		
**Czech Republic**	**5**		5												1						
**Denmark**	**4**		3																		1
**Lithuania**	**4**		4							1											
**Moldova**	**4**		4						1		1			2							
**Norway**	**4**		4																		
**United Kingdom**	**4**		3			2		1											1		
**Austria**	**3**		3																		
**Belgium**	**3**		3																		
**Latvia**	**3**		3	1						1											
**Romania**	**3**		2						1												
**Slovakia**	**3**		3												1						
**Belarus**	**1**		1																		
**Estonia**	**2**		2	2																	
**Hungary**	**2**		2																		
**Serbia**	**2**		2																		
**Spain**	**2**		1							1											
**Sweden**	**2**		2																		
**Finland**	**1**			1																	
**Greece**	**1**															1					
**Luxembourg**	**1**		1																		
**Slovenia**	**1**		1																		
**Ukraine**	**1**		1																		
**Asia**																					
**Russia**	**12**		2	9	2				1							1					
**China**	**7**			3					1			1	3		1					1	
**Japan**	**4**			3			2														
**Mongolia**	**3**			3																	
**Turkey**	**2**		1														1				
**Africa**																					
**Kenya**	**1**																			1	
**Nigeria**	**1**																	1			
**Tunisia**	**1**		1																		

The darker the color the more articles captured.

^a^ Other *Ixodes* spp. with isolated zoonotic *Babesia*: *I*. *spinipalpis*, and *I*. *dentatus* from the United States, *I*. *frontalis* from Moldova, *I*. *simplex* from Romania, *I*. *pavlovskyi* from Russia, and *I*. *granulatus* from China.

^b^ *Haemaphysalis* spp. with isolated zoonotic *Babesia*: *H*. *leporispalustris* from the United States, *H*. *sulcata* from Greece and *H*. *japonica* from Russia.

^c^ *Amblyomma variegatum*.

^d^ *Argas (Carios) vespertilionis*.

^e^ *Rhipicephalus* spp. with isolated zoonotic *Babesia*: *R*. *turanicus*, *R*.*bursa*, and *R*. *sanguineus* from the United States, *R*. *microplus* from China, and *R*. *simus* from Kenya.

*Totals (in grey) do not necessarily equal the sum of corresponding articles (in blue) as articles can report on multiple tick species and/or sample in multiple countries.

### Surveillance

Surveillance to determine the extent of *Babesia* infection in a population was the focus of 29/1394 articles. The majority of the articles were concerned with human infections (23/29). Many relied on passive surveillance wherein physicians report disease cases to state level public health authorities (18/23). Other data collection strategies included active surveillance (3/23) or monitoring programs (2/23). Reports of human babesiosis surveillance were identified from the United States (22/23) and Manitoba, Canada (1/23) [121].

Animal hosts were the subject of active surveillance in 3/29 articles. These reported zoonotic *Babesia* infection in voles [122] and white-footed mice [123] in the United States, as well as seropositive red foxes in Italy [124].

Tick surveillance for *Babesia* was reported in 7/29 articles. European surveillance studies investigated *I*. *ricinus* tick species in Italy [124], and *I*. *ariadnae*, *I*. *vespertilionis*, and *I*. *simplex* in Hungary and Romania [125]. North American studies included 3/7 in the United States [126–128] and 2/7 in Canada [121, 129].

### Treatment

*Babesia* treatments were the focus of 65/1394 articles. Only 2/65 of these articles reported on human treatments. A prospective randomized trial investigated the efficacy of a regimen of atovaquone and azithromycin compared to a regimen of clindamycin and quinine [14]. Another studied the efficacy of atovaquone and cholestyramine combination therapy [130]. Treatments were also investigated in 46/65 animal experiments and 29/65 *in vitro* anti-parasitic experiments. The most common drugs investigated were Imidocarb dipropionate (17/65), Quinine (13/65), Atovaquone (13/65), Diminazene aceturate (12/65), Clindamycin (12/65), Chloroquine (9/65), Pentamidine (8/65), and Azithromycin (6/65).

### Mitigation

Mitigation efforts to prevent and/or control zoonotic *Babesia* were the focus of 94/1394 articles. Interventions for human blood supply safety (20/94), such as screening protocols and tests, were investigated most frequently in the United States (16/20). The impact of pyrethroid-impregnated ear tags for prophylaxis of *B*. *divergens* in cattle was studied in two controlled trials (2/94). Vaccine candidates were explored in 52/94 articles, including 50/52 challenge trials and 3/52 controlled trials using animals. Animal models and *in vitro* studies also examined the impact of adoptive transfer of immunity and various inhibitory chemicals, prophylactic antimicrobials, and microorganisms on *Babesia* or the likelihood of developing *Babesia* infection (20/94).

Preventative control strategies including human behavioural measures, public education to decrease the probability of tick contact for humans and companion animals, landscape modification, and biological control of the vector, were not assessed in any of the zoonotic *Babesia* literature.

### Societal knowledge

Only one article (1/1394) focused on societal knowledge of babesiosis or zoonotic *Babesia* species. Canadian physicians were surveyed to gather information about their knowledge of transfusion-transmitted infection risks and current reporting practices for tick-borne diseases, including babesiosis [131].

### Economic analysis

Seven articles (7/1394) focused on different aspects of the economic impact of babesiosis. One study estimated the median hospitalization costs of babesiosis in Spain [132] and another the costs of tick-borne disease laboratory diagnostic testing in the United States [133]. Cost effectiveness of sampling and laboratory analyses strategies were evaluated in one study using total cost reduction calculations in Italy [134]. The cost-benefit of *B*. *microti* blood donation screening techniques was estimated using quality-adjusted life years in three studies within the United States [135–137]. Another study in the United States evaluated the cost implications of pathogen inactivation for donated platelets [138].

## Discussion

*Babesia* has been prioritized as a disease of public health importance in Canada due to the potential for expansion with climate change. To provide a robust and comprehensive synthesis, we summarized the characteristics of the global evidence on zoonotic *Babesia*. Our intention is that the available literature and knowledge gaps discussed herein will support evidence-informed decision-making on this issue. The findings provide a detailed narrative of the literature available on a global scale, while the discussion has an emphasis on the characteristics, mitigation, and natural transmission of *Babesia* in North America. Since the first case of human babesiosis was reported in 1957, research on zoonotic *Babesia* has steadily increased, particularly under the foci of pathogenesis and epidemiology. However, other important avenues of study such as societal knowledge and economic analyses have seldom been undertaken.

*Babesia microti*, *B*. *divergens*, *B*. *duncani*, and *B*. *venatorum* are among the named species that cause the majority of human cases, and thus are commonly discussed in the literature on babesiosis. Based on their morphology, most of these *Babesia* are sub-categorized as “small *Babesia*” which are closely related to *Theileria* species and include *B*. *microti*, *B*. *duncani*, *B*. *venatorum*, and *B*. *microti-like*, as well as the non-zoonotic species *B*. *gibsoni* and *B*. *rodhaini*. There are also large *Babesia* which consist of *B*. *sp*. *KO1* [139] and the non-zoonotic species *B*. *bovis*, *B*. *cabelli*, *B*. *vogeli*, and *B*. *canis* [2]. *B*. *divergens* is a special case in that microscopically it appears to be a small *Babesia*, but shares a closer genetic lineage to large *Babesia* species [2].

Despite these morphological differences, classification and diagnosis of *Babesia* can present a particular challenge for physicians and laboratory personnel. Microscopically, many species are indistinguishable from each other and are difficult to differentiate from other plasmodial diseases (i.e. malaria). Commercially available serologic tests may have variable sensitivity and specificity and are not reliable for distinguishing between *Babesia* species. Reliably distinguishing between some *Babesia* species that are morphologically and serologically indistinguishable such as *B*. *duncani* and *B*. *microti* requires the use of molecular tests (e.g. polymerase chain reaction) to confidently identify the species [140]. From a public health perspective it is unclear if treatment efficacy differs by species of *Babesia* as currently, treatment depends on the severity of disease rather than species of *Babesia* causing the infection. As vectors and hosts are often shared by a multitude of infectious agents, coinfection with other vector-borne diseases, such as Lyme disease, is not uncommon in areas of endemicity [141, 142]. Coinfections should be considered during diagnosis to ensure appropriate treatment is administered in a timely manner, especially since there is the potential for coinfections to lead to more severe illness [142, 143].

It is also important that physicians are aware of the various routes of human exposure to *Babesia* and collect a thorough patient history regarding recent travel, exposure to ticks, and possible parasite exposure through transfusion or transplants. For example, only 7.2% of Canadian family physicians surveyed in 2002 were aware that babesiosis can be transmitted through blood transfusion [131]. With the incidence of tick-borne disease expected to increase in Canada in the coming years in part due to climate change, an updated survey of both physician and the general population would be helpful in elucidating the current state of knowledge and in guiding future public health promotion and preparedness activities.

There are several common treatments for babesiosis in humans. The majority of mild to moderate babesiosis cases are treatable with combination therapy of atovaquone and azithromycin [14, 130]. Recommendations for treatment of severe babesiosis include the use of clindamycin and quinine combination therapy [15, 144], which is not recommended for treatment of mild to moderate illness due to the risk of adverse reactions [14]. A number of risk factors have been associated with severe *Babesia* infections which may require long or repeated antibiotic therapy [2, 145, 146] and *B*. *divergens* has been associated with severe cases that require adjunct therapies such as red blood cell exchange [147, 148]. Analytic studies investigating the recalcitrance and virulence of *Babesia* species would provide useful evidence for steering treatment decisions. Prescribers and physicians must remain mindful of appropriate treatment regimens that minimize the risk of adverse health risk to patients as well as the potential for antimicrobial drug resistance to arise [149]. Such outcomes could ultimately result in prolonged duration of infection and hospitalization, resulting in increased healthcare expenditure. The economic impact of babesiosis has not been estimated in North America, however an article from Spain calculated the median cost per hospitalized case to be approximately $4710.86 USD [132].

This ScR identified a small number of reports of babesiosis in Canada, which included three human cases designated as locally-acquired [150, 151], three travel acquired cases [152–155], and a transfusion-transmitted case [156]. Babesiosis is not a reportable disease in Canada and only became a reportable disease in 18 of the United States in 2011, 1124 cases were reported that year [5]. However, this disease may currently be underreported as evidence of exposure to *Babesia* based on seroprevalence data from endemic regions of the United States ranges from 6%-16% [157, 158]. In North America, *B*. *microti* is frequently reported in the general immunocompetent population [144], whereas in Europe the majority of clinical cases have been attributed to *B*. *divergens* and have mostly been limited to asplenic individuals [159]. The surveillance data captured out of the United States indicates that there has been an increasing incidence of babesiosis over time [5]; however, like many vector-borne diseases it is unclear whether the rise in reported cases can be attributed to a general increased awareness of the disease, better diagnostic methods, or an actual increased risk of human exposure to *Babesia*. Cohort studies utilizing large sample sizes would be beneficial in gauging risk factors and incidence of infection.

Mitigation efforts in the literature have mainly focused on vaccinations and protecting the blood supply. *Babesia* is considered one of the leading infectious disease threats to blood supplies in the United States, with 165 cases of transfusion-transmitted *B*. *microti* infections reported between 1979 to 2016 [126, 160]. In Canada, there has been only one case of transfusion-transmitted babesiosis, reported in 1999 [156]. No laboratory screening program is in effect for donations in Canada or the United States [161]. Current donor screening and deferral is based upon a self-reported questionnaire question about babesiosis history, which is not a sensitive screening strategy as many infected individuals are asymptomatic [129]. Economic models indicated that donor screening assays may be cost-effective in endemic areas with a high infection prevalence [135, 137]. However, in many areas of the United States screening programs are not considered cost-effective due to the low transmissibility, short acute course, and low mortality associated with *Babesia* infection [136]. In Canada, a seroprevalence survey conducted in 2013 found no positive antibody responses to *B*. *microti* in Canadian blood donors; thus, there was insufficient evidence to warrant a screening program [129]. A second study is underway with 50,000 Canadian blood donors from across the country to provide a more comprehensive and up-to-date risk assessment of *Babesia* in the blood supply [162]. No human behavioural protective measures or environmental interventions such as landscape modification or chemical control measures (e.g. acaricides) were studied for their efficacy in reducing zoonotic babesiosis. However, multiple studies have investigated these interventions for their efficacy in reducing vectors (*I*. *scapularis*) with or without a focus on other pathogens (e.g. lyme disease) [163]. Because these interventions broadly aim to reduce human exposure to these ticks, it is reasonable to conclude they would be effective in reducing the transmission of all pathogens harboured by the targeted tick species, including *Babesia*.

The North American enzootic cycle of *B*. *microti* and the natural transmission between its main vector *I*. *scapularis* and reservoir host, the white footed mouse, has been well documented in the literature [2, 164], as has the natural transmission of *B*. *divergens* between *I*. *ricinus* and bovine hosts in Europe [148, 165]. Other reservoirs of *Babesia* have not been well characterized but may include other rodents, lagomorphs, and cervids [165]. While there has been no evidence of horizontal transmission between animal hosts reported, transplacental transmission has been demonstrated for white-footed mice [46], meadow voles [46] and common voles [47] and may partially explain the high prevalence of *B*. *microti* in endemic sites [46]. This ScR identified evidence confirming the competence of *I*. *scapularis*, *I*. *ricinus*, *I*. *ovatus*, and *I*. *persulcatus* as vectors of *B*. *microti* and/or *B*. *divergens* [8, 53–61, 63–66, 166], which is in agreement with the belief that the majority of vectors of zoonotic *Babesia* species are *Ixodes* ticks [2]. However, for other zoonotic *Babesia* species such as *B*. *duncani*, the reservoir host(s) and tick vector(s) are an area of active research in order to close knowledge gaps [167]. Further epidemiological and experimental research to examine the transmission of these emerging parasites is essential in understanding the enzootic and zoonotic transmission cycles.

The results of this ScR characterize the current evidence and knowledge gaps pertaining to babesiosis and *Babesia* infection. Rigorous efforts were made to capture all relevant literature on the topic; however, there is a possibility that some research evaded identification due to publication bias or absence of indexing. Due to limited translation resources, articles published in any language other than English and French were not characterised in this ScR. This may have resulted in an under-representation of research from some countries which may bias the results of zoonotic *Babesia* research from these areas. The aim of a ScR is to provide an overview of all of the research conducted on a broad topic. A risk of bias of the evidence included is not conducted in a ScR, instead the goal is to identify all relevant research. Thus, the readers should be aware that a wide range of studies is included in the ScR. The field of zoonotic *Babesia* research, including implications for clinical and policy-making practice, would benefit from systematic review(s) of the evidence that address the feasibility and effectiveness of certain treatments, mitigation practices, and resource-using activities such as surveillance and intervention. This ScR has provided an all-encompassing overview of the literature that can help aid in shaping such focused research questions appropriate for systematic review.

There were many evidence gaps identified in this ScR which included few epidemiological studies in certain geographic areas where zoonotic *Babesia* circulate or are likely to emerge. Thus, further epidemiological and surveillance work may be warranted to monitor the potential emergence of *Babesia* into previously unaffected areas. Keeping apprised of the emergence and burden of *Babesia*, diagnosis, treatment, and effective mitigation strategies is important to inform future policy and decision making on this issue and guide public health mitigation programs.

## Supporting information

S1 Protocol Protocol and search strategies employed for our scoping review on the characteristics of the global evidence of babesiosis and zoonotic *Babesia*. (DOCX)

S1 Dataset List of references and full data characterization dataset analyzed in our scoping review on the characteristics of the global evidence of babesiosis and zoonotic *Babesia*. (XLSX)

S1 Exclusions References excluded from our scoping review due to language. (XLSX)

S1 Table Table of host to vector and vector to host *Babesia* transmission scenarios captured in our scoping review. (DOCX)

S2 Table Table of animal epidemiology outcomes and sporadic case reports captured in our scoping review. (DOCX)
