# Supplementary material for: Zoonotic Babesia: A scoping review of the global evidence
Source: PLoS One. 2019 Dec 30;14(12):e0226781. doi: 10.1371/journal.pone.0226781 (PMC6936817; doi:10.1371/journal.pone.0226781)
Supplement: S1 Protocol — (DOCX) [file pone.0226781.s001.docx]

# S1 Protocol. Protocol for Scoping Review of Babesia

# Title:

**What are the characteristics of the global evidence on Babesiosis and *Babesia* sp. that infect humans?**

Authors:

Kaitlin M. Young^1^, Tricia Corrin^1^, Barbara Wilhelm^2^, Carl Uhland^3^, Judy Greig^1^, Mariola Mascarenhas^1^, Lisa A. Waddell^1^

^1^Public Health Risk Sciences Division of the National Microbiology Laboratory, Public Health Agency of Canada, Guelph, Ontario, Canada

^2^Big Sky Health Analytics, Vermilion, Alberta, Canada

^3^Independent Consultant, St-Hyacinthe, Quebec, Canada

Contact: Tricia Corrin, Tel: 226-979-7174 or email: patricia.corrin@canada.ca

## Important Dates:

Evidence published up to February 20, 2017

Protocol version 1, initiated February 14, 2017

Protocol search results updated June 7, 2018

Contents

[Rationale 2](#_Toc466890315)

[Background 2](#_Toc466890316)

[Study Question 5](#_Toc466890317)

[Planned Study Outputs 5](#_Toc466890318)

[Methods 5](#_Toc466890319)

[Review Team Expertise and Responsibilities. 5](#_Toc466890320)

[Search Strategy 5](#_Toc466890321)

[Algorithms 5](#_Toc466890322)

[Databases 6](#_Toc466890323)

[Grey Literature Sources and Procedures 6](#_Toc466890324)

[Search Verification 6](#_Toc466890325)

[Relevance Screening (RS) 6](#_Toc466890326)

[Inclusion / Exclusion criteria 6](#_Toc466890327)

[Study Characterization 6](#_Toc466890328)

[Review Management 7](#_Toc466890329)

[Data Analysis 7](#_Toc466890330)

[Appendix 1: Relevance Screening Tool 8](#_Toc466890331)

[Appendix 2: Data Characterization and Utility (DCU) form 9](#_Toc466890332)

[Appendix 3: Search Strategy Implemented 25](#_Toc466890333)

## Rationale

This research aligns with PHRSD priorities, which include enhancing and guiding public health decision-making and policies by providing the authoritative analyses, recommendations and scientific collaborative services (using methods such as epidemiological studies and knowledge synthesis) to address the occurrence, trend and determinants of infectious disease in Canada with expert focus on the prevention of public health risks arising from the food chain, animals and the environment (LFZ, 2013).

This project has been prioritized via stakeholder consultation as an important vector-borne disease (VBD) that is likely to expand its range in Canada due to climate change. Funding for this project has been provided by the Public Health Agency of Canada’s VBD climate change and adaptation funding 2016-2021).

## Background

Babesiosis in humans is caused by microscopic parasites called *Babesia* sp. from the phylum Apicomplexa, in North America, Australia, Canary Islands, and Asia (Japan and Taiwan). Most human infections are due to *Babesia microti* that infect red blood cells. Babesiosis occurs in Europe and sporadically through the rest of the world. There are over 100 species of *Babesia* (phylogenetic tree: <http://www.onezoom.org/AT/@=202828?vis=spiral#x1729,y133,w0.8614> )*,* however only a few have occasionally been found in people. In addition to *B. microti*;

- *B. divergens* (human parasite cases in Kentucky, Missouri, Washington and in Europe transmitted by *I. ricinus*)
- *B. duncani* (aka unnamed CA1) (cattle parasite also found in humans in Europe/Australia/Pacific coast California to Washington),
- *B. venatorum* (aka unnamed Babesia EU 1-3 & B. divergens-like species) (Roe deer parasite in Europe- Austria, Germany, Italy),
- large *Babesia* (>3um) (infects ungulates)
- Unnamed agent (humans in east Africa and South America – may not be the same.)
- **Others**: *B. microti -like (Japan, Taiwan and Europe),* B. duncani-type organisms, Babesia sp. K01 (from S. Korea), Babesia sp. MO1 (unnamed), Babesia sp. WA1 (unnamed).

*Babesia* is considered the second-most common blood parasite of mammals, which has an impact on domestic animals (cattle) in areas without severe winters.

*Babesia microti* is transmitted by *Ixodes scapularis* ticks usually in the nymph stage (size of a poppy seed). The transmission cycle for *Babesia microti* includes animal hosts such as the white-footed mouse and other small mammals (see life cycle below). Most cases occur during warmer months (spring and summer) in wooded areas, brush or grass, as this is when nymphs are most active. *I. scapularis* nymphs need to stay attached to a human for more than 36-48 hours to transmit *B. microti*.


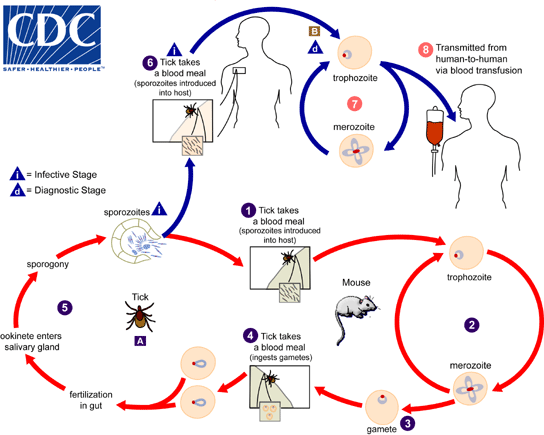


The *Babesia microti* life cycle involves two hosts, which include a rodent, primarily the white-footed mouse, *Peromyscus leucopus,* and a tick in the genus *Ixodes*. During a blood meal, a *Babesia*-infected tick introduces sporozoites into the mouse host 
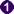
. Sporozoites enter erythrocytes and undergo asexual reproduction (budding) 
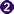
. In the blood, some parasites differentiate into male and female gametes, although these cannot be distinguished by light microscopy 
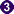
. The definitive host is the tick. Once ingested by an appropriate tick 
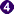
, gametes unite and undergo a sporogonic cycle resulting in sporozoites 
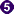
. Transovarial transmission (also known as vertical, or hereditary, transmission) has been documented for "large" *Babesia* species but not for the "small" *Babesia*, such as *B. microti* 
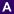
.

Humans enter the cycle when bitten by infected ticks. During a blood meal, a *Babesia*-infected tick introduces sporozoites into the human host 
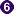
. Sporozoites enter erythrocytes 
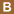
 and undergo asexual replication (budding) 
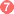
. Multiplication of the blood-stage parasites is responsible for the clinical manifestations of the disease. Humans usually are dead-end hosts. However, human-to-human transmission is well recognized to occur via contaminated blood transfusions 
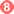
. (From CDC)

*B. microti* has also been shown to transmit human to human by transfusion of contaminated blood (there is no screening test for blood donors) and from an infected mother to her baby during pregnancy/delivery, however both transmission routes are rare. It can take a week (usually 1-4 weeks after tick bite or 1-9 after blood transfusion) to months after exposure to *Babesia* sp. to develop symptoms and because the nymphs are so small, most people do not recall a tick bite.

In North America babesiosis has been recorded in the Northeast and upper Midwest USA (New England, New York, New Jersey, Wisconsin and Minnesota). Babesiosis is a reportable disease in some states and in 2011 a surveillance program was initiated with 18 USA states (California, Connecticut, Delaware, Indiana, Maine, Maryland, Massachusetts, Minnesota, Nebraska, New Hampshire, New Jersey, New York, Oregon, Rhode Island, Tennessee, Vermont, Washington, Wisconsin) and 1 city (New York city) (1124 cases were reported in 2011).

In Canada, the first locally acquired case was in 2013 in Manitoba and was detected in *I. scapularis* in Manitoba since 2010 as well as in rodent populations on two occasions. A recent study found infected ticks in Manitoba (1.7%), Ontario (1 positive passive submission), and Quebec (0.5%); however there was no infection in blood donors during the same time period.

Infections with Babesia in humans can range from asymptomatic to life threatening. It is thought that half of infected children and a quarter of healthy adults are asymptomatic. Mild symptoms include flu-like symptoms: fever, chills, sweats, headache, body ache, loss of appetite, nausea, fatigue, and headache. Less common symptoms include: muscle pain,anorexia, nonproductive cough (mucus is not coughed up), arthralgias (noninflammatory joint pain, unlike arthritis, which is inflammatory), vomiting, sore throat, abdominal pain, pink eye, photophobia (abnormal intolerance to visual perception of light), weight loss, emotional lability, depression , hyperesthesia (more sensitive to stimuli), pharyngeal erythema, retinopathy with splinter hemorrhages, retinal infarcts, and neutropenia. *Babesia* can also cause hemolytic anemia due to the destruction of red blood cells; symptoms include jaundice and dark urine. Other complications include: unstable blood pressure, low platelet count, disseminated intravascular coagulation (DIC) or consumptive coagulopathy (clots/bleeding), malfunction of vital organs (splenomegaly, hepatomegaly) or death. Populations at high risk of severe disease include people without a spleen, immunocompromised, and elderly.

Diagnosis includes microscopic examination of red blood cells for *Babesia sp.;* it can be difficult to distinguish between Babesia and Plasmodium (especially P. falciparum) parasites and even between parasites and artifacts (such as stain e.g. Giemsa, Diff-Quick, Romanowsky, Field’s or modified Wright’s stain or platelet debris). However, identification of Babesia in stained erythrocytes is considered a definitive diagnosis. The light microscope detection limit is 0.001% parasitaemia (~ 5000 infected erythrocytes/ ml). So-called "Maltese cross formations" on the blood film are indicative of *Babesia* sp. infection, since they are not seen in malaria, the primary differential diagnosis. But more often simple rings (annular) and paired or single pear-shaped trophozoites (pyriform) are seen. Careful examination of multiple smears may be necessary as this method has low sensitivity, since Babesia may infect less than 1% of circulating red blood cells and therefore can easily be overlooked. To aid in higher density samples, it is suggested to draw them from peripherial capillaries like the ear and nail beds. Babesia can be detectable in the blood for 3 weeks to 12 weeks (or up to 7 months in rare cases). This may be confirmed by a specialized laboratory—by blood-smear examination and, if indicated, by other means, such as molecular and/or serologic methods tailored to the setting/species. Serologic testing doesn’t seem to be conducted much in North America, but it exists. For antibodies against *Babesia* (both IgG and IgM) can be detected in cases where there may be a low-level of infection and negative blood film examinations. Serology is also useful for differentiating babesiosis from malaria in cases where people are at risk for both infections. Since detectable antibody responses require about a week after infection to develop, serologic testing may be falsely negative early in the disease course. Indirect fluorescent antibody test (IFAT) has superior specificity, ELISA & dot-ELISA have superior sensitivity and newer recombinant ELISAs have improved Specificity making them the better choice. Due to cross-reactivity between babesia sp, serology cannot easily determine the Babesia species and high sero-reactivity has been noted in endemic areas. A polymerase chain reaction (PCR) test has been developed for the detection of Babesia from the peripheral blood. PCR may be at least as sensitive and specific as blood-film examination in diagnosing babesiosis, though it is also significantly more expensive. Most often, PCR testing is used in conjunction with blood film examination and possibly serologic testing. Nested PCR, real-time PCR, reverse line blotting techniques. More sensitive (detection limit 50 or/ml) than blood smear and can id species, but is negative with

Infection is treatable, but no vaccine exists. For ill patients, babesiosis usually is treated for at least 7-10 days with a combination of two prescription medications — typically either: atovaquone plus azithromycin; OR clindamycin plus quinine (this combination is the standard of care for severely ill patients). Patients with relapsing disease can be treated for 6 weeks. Other treatments may be required as supportive care for the symptoms of babesiosis: antipyretics, vasoprssors, blood transfusion, mechanical ventilation, dialysis.

Prevention of babesiosis and other tickborne infections include tick avoidance and personal protective measures such as repellants, protective clothing and tick checks.

**Sources:**

Babesiosis: accessed February 14, 2017, <https://www.cdc.gov/parasites/babesiosis/>

Babesiosis: accessed February 14, 2017, <https://en.wikipedia.org/wiki/Babesiosis>

Obrien et al. Seroprevalence of Babesia microti infection in Canadian blood donors. Transfusion. 2016 Jan;56(1):237-43. doi: 10.1111/trf.13339.

Homer et al. Babesiosis. Clinical microbiology reviews. 2000: 13,(3) 451-469.

Beugnet and Moreau. Babesiosis. Rev. Sci. Tech. Off. Int. Epiz. 2015: 34 (2) 627-639.

Bannier. Human babesiosis. N Eng J Med. 2012: 366:25: 2397 – 2407.

##

## Study Question

**What are the characteristics of the global evidence on Babesiosis and *Babesia* sp. that infect humans?**

## Planned Study Outputs

1. A scoping review of the global evidence.
2. A repository and dataset of all relevant literature captured in this study.

# Methods

## Review Team Expertise and Responsibilities.

| **Member** | **Organization** | **Project Role*** |
| --- | --- | --- |
| Lisa Waddell | PHRS /NML | Synthesis expertise/oversight/reveiwer |
| Judy Greig | PHRS /NML | Synthesis expertise/oversight/reveiwer |
| Mariola Mascarenhas | PHRS /NML | Synthesis expertise/reviewer |
| Kaitlin Young | PHRS /NML | Co-lead/synthesis expertise/reviewer |
| Barbara Wilhelm | Big Sky Consulting | Synthesis expertise/reviewer |
| Carl Uhland | Consultant | Synthesis expertise/reviewer |
| Tricia Corrin | PHRS /NML | Co-lead/Synthesis expertise/reviewer |

## Search Strategy

### Algorithms

(babesiosis and (human or humans or man or woman or people))or ( babesia)

Endnote database- BabesiaCitations_Feb2017

### Databases

- Scopus TITLE-ABS-KEY (hits=), PubMed/MEDLINE all fields and MESH( ), Embase all field ( ), Global Health all field ( ), COCHRANE library for any relevant trials in the trial registry ()
- Proquest theses and dissertations (for grey literature)

### Grey Literature Sources and Procedures

To augment the electronic database search already performed, the following steps will be taken to execute a search of the relevant grey literature:

1. A complementary search for grey literature documents (e.g. conference proceedings and research reports) will be conducted in Google using the algorithms employed in the electronic search. The first 100 Google hits will be investigated using a ‘snowball’ approach, to identify relevant websites, which will then be investigated for relevant literature (to be identified using the RS1 screening question).

2. The following jurisdictions will be investigated for additional relevant surveillance data:

i. Canada: Federally and individual provinces, using Google search, employing the same search terms and algorithm as the initial electronic database search, and a similar ‘snowball’ search examining the first 100 hits. The project topic expert indicated that to his knowledge no Canadian data were available other than that already captured from peer reviewed literature by the initial electronic search.

ii. US: Federally and individual states, using CDC website, and Google search, employing the same search terms and algorithm as described above.

Additionally, for Canada and the U.S., states/provinces from which the review has captured reports of human Babesia cases will be the subject of online searches for notification/monitoring system

ii. Europe: using CDC Europe website, and Google search for individual countries captured by the initial electronic database search.

iv. Asia: CDC Asia website, and Google search of countries captured by initial electronic database search.

v. South America: PAHO website, and Google search of countries captured by initial electronic database search.

3. Specialized electronic bibliographic databases capturing grey literature (OpenGrey (http://www.opengrey.eu/) and DataCite (https://search.datacite.org/) will also be searched.

4. Google searches are to be conducted individually, for each of the countries from which research has been previously captured by the bibliographic database search, in two separate forms. For each country, a Google search will be conducted combining (Country) AND (Babesia). Additionally, a Google search will be conducted for ministries of health or public health, and where possible, searched for Babesia literature.

As the grey literature search will be conducted by an English/French researcher, and articles ultimately selected for this review are limited to those published in English and French languages only, there is possibility of language bias.

### Search Verification

Ten literature reviews or key articles focused or partially focused on babesiosis in humans will be identified. Reference lists will be screened for potentially relevant citations missed by the electronic search. At the point of saturation, when no new references are identified, we will stop evaluating reference lists.

Papers evaluated:

1. Homer, M. J., et al. (2000). "Babesiosis." Clinical Microbiology Reviews 13(3): 451-469.
2. Ord, R. L. and C. A. Lobo (2015). "Human Babesiosis: Pathogens, Prevalence, Diagnosis and Treatment." Current Clinical Microbiology Reports 2(4): 173-181.
3. Usmani-Brown, S., et al. (2013). "Neurological manifestations of human babesiosis." Handb Clin Neurol 114: 199-203.
4. Dunn, I. J. and P. E. Palmer (1998). "Babesiosis." Semin Roentgenol 33(1): 89-90.
5. Spielman, A., et al. (1985). "Ecology of Ixodes dammini-borne human babesiosis and Lyme disease." Annu Rev Entomol 30: 439-460.
6. Rosner, F., et al. (1984). "Babesiosis in splenectomized adults. Review of 22 reported cases." Am J Med 76(4): 696-701.
7. Ruebush Ii, T. K. (1980). "Human babesiosis in North America." Transactions of the Royal Society of Tropical Medicine and Hygiene 74(2): 149-152.
8. Lempereur, L., et al. (2017). "Guidelines for the Detection of Babesia and Theileria Parasites." Vector Borne Zoonotic Dis 17(1): 51-65.
9. Gelfand, J. A. and M. V. Callahan (2003). "Babesiosis: An Update on Epidemiology and Treatment." Curr Infect Dis Rep 5(1): 53-58.
10. Gray, J. S. (2006). "Identity of the causal agents of human babesiosis in Europe." Int J Med Microbiol 296 Suppl 40: 131-136.

## Relevance Screening (RS)

The relevance screening level will be done on the title, abstract, and keywords where available. There is 1 question that encompassed the inclusion / exclusion criteria, this tool can be found in the appendix.

### Inclusion / Exclusion criteria

1. Time frame – no time frame
2. Country – All
3. Language – English, French. All other languages will be identified and parked until resources and time is available. e.g. Spanish or Portuguese
4. Document Type: journal articles, PhD/MSc Theses, reports, conference papers.

5) Agent/Disease: Babesia (all species pathogenic to humans)

6) Study design: all

7) Primary research: all articles describing primary research on the pathogen, any reservoir, vector or incidental host will be included. Relevant secondary research e.g. literature reviews and predictive models will be identified as such for search verification unless they are evaluating the impact of climate change on this pathogen, in which case it will be included for summarization. All other citations will be excluded.

### Evaluation of Babesia sp. Pathogenic to humans – pre DCU analysis-

R1 Tool included: *B. microti*, *B. divergens, B. duncani,* *B. venatorum*, large *Babesia* (>3um) if noted to be associated with human infection, Unnamed agents in humans and any article that referenced zoonotic Babesia.

1. Reviewers noted the following issues while relevance screening.

- Babesia sensu lato refers to a group of Babesia that includes B. canis, B. divergens and B. rossi. Thus, we may need to reclassify this subset into the include box. Also note, B. canis is excluded at this point as a non-zoonotic Babesia, however several studies note that it is very closely related to B. divergens.
- Studies with NO speciation even with the full paper. Exclude unless the sample population or source of an isolate was human.
- Data characterization ONLY on the included Babesia. The risk is that we get half way through the DCU and need to include a Babesia we had not identified as zoonotic. Solution: reviewers will check off the included Babesia from a list of babesia that infect humans and extract data on them, but they will also check off the Babesia in the article that are not known to be zoonotic (under the second part of the relevance question) so we know they are present in the paper, but we will not extract data on the animal only babesia.

## Study Characterization

The study characterization form will aim to classify and characterize the research on babesiosis in humans and all research on *Babesia* sp. that can infect humans so we can understand where there are areas of knowledge saturation and gaps. The form will first confirm the relevance of the publication prior to extracting important characteristics of the study. This will include; study design, population, setting, outcomes, whether there is extractable data, and if the study addresses the impacts of climate change on the parasite.

## Review Management

The search strategy will be compiled and de-duplicated in an Endnote database. This database will then be exported to DistillerSR, a web-based systematic review software designed to manage all stages of conducting scoping reviews and systematic reviews. All stages of the scoping study from relevance screening to data extraction will be conducted within this software. The final dataset will be exported into MS Excel, cleaned and tabulated for use in the publication and reports.

## Data Analysis

Descriptive tabulation of all pertinent information that aids in the characterisation and illustration of the available knowledge on Babesiosis will be conducted mainly in MS excel unless further statistical analysis is required. Findings and recommendations, methods incorporated and their usefulness, and study limitations will also be captured.

## Appendix 1: Relevance Screening Tool

**What are the characteristics of the global evidence on Babesiosis and *Babesia* sp. that infect humans?**

**Relevance Screening Tool for Abstracts:**

| **Question** | **Options** | **Definitions/additional notes** |
| --- | --- | --- |
| Does this citation describe primary research on **Babesiosis in humans or** ***Babesia sp.* that is pathogenic (can infect) to humans** (in vectors, hosts, humans or a parasite only study) or a predictive model examining the impacts of climate change on Babesia/Babesiosis? |  Yes – relevant primary research   Yes- relevant predictive CC model   - No – primary research on “other *Babesia sp.*” that is NOT associated with disease in humans.   *Please check off or add the parasite species to this list:*   No – relevant review   No – conference proceeding book   No – book, not primary lit.   No – other relevant non-primary source   No, not relevant (excluded, submit form) | **Babesia sp:** is a small parasite that infects red blood cells. There are >100 species, but only a few are recognised to be pathogenic to humans: B. microti, *B. divergens, B. duncani* , *B. venatorum*  **Babesiosis:** is an Infection of the red blood cells with *Babesia sp.* in humans. Disease can range from asymptomatic to life threatening. It is though that half of infected children and a quarter of healthy adults are asymptomatic  **Primary research** represents a study where the authors collected and analyzed their own data – may use quantitative or qualitative methods or both to investigate the research question and report original results.  **Predictive Model:** Any citation describing the use of published information to model the issue and make predictions. **Climate change:** any predictive model looking at the effects of a changing climate e.g. temperature, rainfall etc. should be included in this review.  **Review/commentary** is a comprehensive or brief narrative review or commentary (from peer-reviewed articles journals) summarising knowledge on an issue (include systematic reviews in here.)  **Other non-primary** will encompass lay magazine or newspaper articles etc.  Exclude:  Primary research not on the parasite of interest. |

2 reviewers independently will evaluate each citation.

## Appendix 2: Data Characterization and Utility (DCU) form

**Broad topic:**

**What are the characteristics of the global evidence on Babesiosis and *Babesia* sp. that infect humans?**

We are only interested in characterizing the literature on Babesia that can infect humans and ALL literature on Babesia in humans. Babesia that we know infects humans (may not be exhausted) includes: B. microti, *B. divergens, B. duncani* (aka unnamed CA1), *B. venatorum, B. microti -like (Japan, Taiwan and Europe),* B. duncani-type organisms, B. venatorum (aka unnamed Babesia EU 1-3 & B. divergens-like species), B. odocoilei, Babesia cf. odocoilei, Babesia sp. K01 (from S. Korea), Babesia sp. MO1 (unnamed), Babesia sp. WA1 (unnamed). See the Taxonomy xls to find the Babesia species and unnamed Babesia (zoonotic ones are highlighted in yellow).

**Note:** Remember to only extract information for the applicable question – **not all questions apply.** Be very specific about the data you extract AND only **extract primary information** (information collected by the author in the course of a study).

| **Question** | **Options** | | **Definitions/Additional notes** | |  |
| --- | --- | --- | --- | --- | --- |
| **Relevance Verification**  The first four questions are designed for verification of the relevance of the article. Please answer all 4 questions if the article is **primary research** even if it is in a foreign language as we would like to characterize the foreign language papers by focus of the paper. If the foreign language papers is likely not relevant, please indicate this in the focus question so we are not over inflating the number of relevant studies we excluded due to language.  **Non-primary** research e.g. lit reviews can be selected at the second question and the form can be submitted. | | | | |  |
| What language is the article published in? | - English - French - Other, please specify: ____ (Exclude) | |  | |  |
| What type of document is this article? | - Primary research in peer-reviewed journal - Predictive model - Thesis - Grey literature with primary data (government or research reports) - Conference proceeding with sufficient detail - Literature review (Exclude) - Systematic review/meta-analyses (Exclude) - Grey literature; may report previously reported research (e.g. newspaper or magazine articles) (exclude) - Conference proceeding with insufficient detail (exclude) | | **Primary research:** original research/investigation/study carried out by the researcher (incl. surveys, interviews, outbreak reports, observations, etc.)  **Thesis:** a long paper/essay or dissertation involving personal research (usually written for a university degree)  **Conference proceeding abstract/short paper:** a collection of published academic papers  **Literature review:** examination of published literature  **Systematic review/meta-analyses:** analysis and interpretation of primary research  **Grey literature:** research that is unpublished or published in a non-commercial form | |  |
| Determine what *Babesia sp.* are studied or isolated in this publication (if only serology is conducted and it does not target a specific babesia species please check the serology option) and whether it is a *Babesia sp.*  that causes disease in humans or is not known to infect humans:  *(When adding options please note if it is an un-named Babesia- see taxonomy spreadsheet for list.*  *Fully answer this question for* ***ALL Babesia*** *in the study, however if only Babesia NOT known to cause disease in humans are selected, submit the form after the next question.)* | ***Babesia sp. that infect humans***   - *Babesia sp.* not determined, but IS isolated from humans - **Only serology** conducted on human samples, Babesia species not specified. - No babesia spp. found in this study, but would have been speciated if identified. (exclude) - Not applicable (no *Babesia* sampled in this study) - *B. microti* - *B. divergens* - *B. duncani* - *B. venatorum* (includes unnamed EU 1-3) - B. odocoilei - B. cf microti (microti-like) - B. duncani-like - B. divergens-like - B. cf. odocoilei - Babesia sp. KO1 - Babesia sp. MO1 (unnamed) - Babesia sp. WA1 (unnamed) - Add as needed. (list will be updated after R1)   ***Babesia sp. that are not known to infect humans (exclude article if ONLY Babesia from this list are checked)***   - *Babesia* sp not determined AND is NOT isolated from humans. - Only serology conducted, babesia species not specified and samples were NOT from humans. - Add as needed. (list will be updated after R1) | | See introduction for an explanation of the different *Babesia sp.*  The species listed here are known to cause disease in humans in decreasing order.  There are >100 species of *Babesia* and most do NOT infect humans. So please put all Babesia identified in human samples, identified as being zoonotic or are known to infect humans (see list) under the human list.  All Babesia that are not known to infect humans can go under the options in the second list.  NEGATIVE RESULTS STUDIES IN ANIMALS WHERE BABESIA TARGET SPECIES IS NOT SPECIFIED  - They looked for / screened for a **not determined** Babesia sp. in animals. Depending on the diagnosis method there are 2 options:  using virus isolation/PCR = 1^st^ option “*Babesia* sp not determined AND is NOT isolated from humans.”,  using serology = 2^nd^ option “Only serology conducted, babesia species not specified and samples were NOT from humans” | |  |
| Verify the relevance and focus of the paper. This paper describes research on Babesiosis or *Babesia sp*. that can infect humans or describes a climate change model for Babesiosis/*Babesia* sp.?  (Check all that apply; When answering this question, only check off the topics for which there are study outcomes and do not check if a category was just “mentioned” in the paper.) | - **Pathogenesis** of relevant *Babesia* sp. in humans and/or animal hosts - *Babesia sp.* infection, diagnosis, clinical characteristics and complications including affected organs and systems in any species - Infection mechanism (cellular level) in the host - Immune response in the host (proteins, genes and receptors) - Animal pathogenesis model - **Investigation of Babesia Treatments** - **Diagnostic test** evaluation for *Babesia sp.* - **Epidemiology** of Babesiosis or relevant *Babesia sp.* (prevalence, incidence, risk factors of exposure/disease) - **Transmission/Parasite competence:** conditions for transmission of *Babesia sp.* between vector and animal host or human. OR studies on vector characteristics/ behaviour for *Babesia sp.* transmission). - **Surveillance** to determine the extent of Babesiosis or *Babesia sp.* infections - ***Babesia sp.* study** (Examines the parasite attributes such as pathogenesis, transmission characteristics, and/or molecular characterisation) - **Mitigation/Interventions** to prevent and/or control *Babesia sp.* infection in humans, hosts or vectors. - **Societal knowledge, attitudes and/or risk perceptions** towards Babesiosis or *Babesia sp.* and potential mitigation strategies - **Economic burden or cost-benefit** analysis of Babesiosis or *Babesia sp.* infection and/or mitigation strategies - **Predictive model** on the impact of climate change on Babesiosis or *Babesia sp.* - **Other** Babesiosis or *Babesia sp.* topic (Use if absolutely necessary) - Not relevant to the review including research on predictive models for the vector and research on vectors of *Babesi*a sp., but not the parasite itself (e.g. mitigation, abundance, density, general survival attributes and characteristics of the vector unrelated to its B*abesia sp.* status.) | | ***Pathogenesis:*** biological processes/mechanisms/  pathways that lead to Babesiosis in human, or animal hosts. This includes the following:   - Pathology of disease (chronic or acute signs and symptoms and organs /systems affected e.g. CNS) - Infection mechanisms (at cellular level, stages of infection) including *Babesia sp.* entry/exit or inhibitors of *Babesia sp.* entry/exit in the host, entry, replication and clearance. - Immune response (Proteins/genes/receptors involved; in host and vector) - Animal models studying pathogenesis (Check in addition to one of the above options if animal model is used.)   ***Treatments:*** Only include studies that examine the effectiveness of Babesia treatments. Do NOT record how cases were treated for Babesia.  ***Diagnostic tests*** refer to tests detecting the presence of *Babesia sp.* in humans, non-human hosts or vectors.  ***Epidemiology:*** Please include articles describing outbreak and sporadic cases, incidence/ prevalence for Babesiosis or *Babesia sp.* infection, and/or risk factors for developing *Babesia sp.* infection or risk factors/conditions (environmental and climatic mostly) for *Babesia sp.* survival in vectors.  *Risk factors* are environmental, behavioural, or biologic factors usually in longitudinal, cross-sectional, cohort or case control studies where exposures and outcomes are studied. A risk factor indicates an association with an increase or decrease in disease in the population with the risk factor compared to that without.  ***Transmission*** passing of the parasite from an infected host to another vector/host; e.g. tick transmission, mother to child and/or through blood transfusion and adaptability, the ability to adapt to new host/environment or become resistant to drug. This could also be climatic conditions required for transmission.  ***Parasite competence*** include:   - Characteristics of competent *Babesia sp.* vector (genes, adaptations, etc...; ability to transmit disease) - Range and density of *Babesia sp.* infected vector and/or environmental/climatic conditions to sustain *Babesia sp.* infected vector population - Vector activity (biting rate, Fecundity/fertility rate, reproductive rate etc.) - Extrinsic incubation period (Interval between the uptake of *Babesia sp.* by vector and vector’s ability to transmit *Babesia sp.* to other susceptible hosts) - Transmission/rate of infectivity (ie: how many people could be exposed by one infected vector and how many vectors are likely to become infected by one infected human or animal/host)   ***Surveillance*** is the ongoing and systematic collection, analysis, and interpretation of outcome-specific data for use in the planning, implementation, and evaluation of public health practice. Include studies evaluating surveillance methods/programs.  Examples :   - Surveillance of human cases - Sylvatic host surveillance (Sylvatic cycling is when pathogen transmission occurs between animal i.e., sylvatic hosts and vectors) - Mosquito/vector surveillance   ***Babesia sp. studies*** are typically studies that focus only on the parasite with no affiliation to a host, thus *in vitro* experiments or analysis is focused on genetic analysis to characterize the parasite.   - Molecular characterization of *Babesia sp.* (e.g. mutations, phylogenetic analysis) - Phylogenetic trees (only if there is one reported in the paper! - Whole genome sequencing (WGS) check this off if they did it in the study. - *Babesia sp.* pathogenic attributes (Describes how parasite causes disease in the host e.g. virulence factors, parasite entry/exit/cycle (includes latency period), replication)   ***Mitigation / interventions***  *Relevant mitigation studies need to have an outcome that measures a change in the burden of Babesia sp. or babesiosis in humans, hosts or vectors. Measuring only abundance of ticks (or other vector) is outside the scope of this review and will be captured in the vector mitigation reviews.*   - Studies looking at intervention efficacy include control or challenge trials and quasi experiments (before and after). - Program evaluations can fall in here. - Risk factors looking at presence/absence of an intervention should also be checked here. - Examples include (but are not limited to) land management, vector management and control, personal protection, and public education campaigns   ***Risk perceptions*** are the subjective judgements that people make about the characteristics and severity of a risk. Do individuals feel they are at risk? Do they have knowledge that they can implement to decrease their risk? What are their feelings concerning using sprays or treating vectors to decrease the risk of disease transmission?  ***Economic burden*** will include an actual dollar amount or discussion of implied cost associated with mitigation strategies.  ***Cost benefit analysis*** is a systematic process for calculating and comparing benefits and costs of a project, decision or government policy.  ***Predictive models*** are mathematical or statistical models used to forecast outcomes, spread of *Babesia sp.* and/or trends. Examples include (but are not limited to) using climate to predict outbreaks and/or models predicting high-risk populations. In the provided text box, please describe model in one line. If possible, copy and paste text from the abstract/objectives section. | |  |
| **If exclusion criteria were selected above, submit the form before proceeding** | | | | |  |
| **General Information**  ONLY ANSWER THE DCU FORM FOR THE BABESIA THAT ARE KNOWN TO CAUSE DISEASE IN HUMANS – we are not interested in the data for the other babesia. | | | | |  |
| From what continent(s) were the samples obtained? (If not specified, resort to author affiliations)  Specify the country(ies)  If this is an observational study specify the US state or Canadian province: | - North America - Europe - Australasia - Central America/South America/Caribbean - Asia - Africa - Other - Canada - USA - Add options as they occur   Provinces   - AB - BC - MB - NB - NL - NS - NT - NU - ON - PE - QC - SK - YT   States   - California - Connecticut - Delaware - Indiana - Maine - Maryland - Massachusetts - Minnesota - Nebraska - New Hampshire - New Jersey - New York state - New York city - Oregon - Rhode Island - Tennessee - Vermont - Washington - Wisconsin - Add options as they occur | | **North America:** includes Canada, USA and Mexico  **Europe:** includes, Belarus, Latvia, Ukraine, Estonia, Cyprus & west (incl. Iceland and Greenland)  **Australasia:** limited to Australia, New Guinea, New Zealand, New Caledonia, and neighbouring islands, including the Indonesian islands from Lombok and Sulawesi eastward  **Central America/South America/ Caribbean:** includes Caribbean, and all of south and central America.  **Asia:** Russia, Turkey, middle eastern countries and east  **Please select or add the country to the list**  **ONLY for observational studies,** indicate the Canadian province or US state the sampling frame was drawn from. | |  |
| When was the article published*? Specify year XXXX* | ___TXT___ | | **Year** e.g. 1979 | |  |
| When were samples collected or the study conducted? *Specify year XXXX* | ___TXT___ | | **Dates**: year/month (if available) e.g. 1984/05 – 1989/12  Note “NA” for experiments unless actually specified. | |  |
| What is the study design? | - Observational study - Case series/ case report - Population-based case series - Cohort - Case control - Cross-sectional - Prevalence survey - Surveillance or monitoring program - Outbreak investigation - Longitudinal study - Other OBS: ___ - Experimental study - Controlled Trial - Challenge trial - Quasi-experiment - Molecular characterization - Other EXP:___ - Evaluation of a diagnostic tests - Molecular epidemiology - Qualitative research - Predictive model - Economic model - Risk assessment - Other: specify ____ | | **Observational study:** Assignment of subjects into treated group versus a control group is outside the control of the investigator.  **Case series/report:**  an in depth evaluation of one or more cases and their clinical history/ risk factors.  **Population-based case series**: Often the findings of a disease surveillance program where the results represent disease in a geographical area.  **Cohort:** prospectively follow a group of exposed and non-exposed individuals to evaluate whether they develop an outcome or retrospectively evaluate exposure / disease when the exposure was likely to be a point source such as a foodborne outbreak at a wedding.  **Case control:** usually retrospective, identified cases are matched with controls and their risk factors are evaluated for an association with disease.  **Cross-sectional:** Examines the relationship of a risk factors and outcome (disease) at a point in time on a representative sample of the target population.  **Prevalence survey:** A measurement of the outcome (disease)at a point in time on a representative sample of the target population.  **Surveillance/Monitoring program results**: on-going sampling from a defined representative sample of the target population to evaluate changes over time.  **Controlled Trial**: experiments where the investigator has control over the experiment, they ideally randomize subjects into treated and non-treated groups and apply uniform measurements of the outcome.  **Challenge trial** is a controlled trial that includes exposure to the agent  **Molecular characterization**: This is an experimental study evaluating functional attributes of the pathogen, developing phylogenetic trees from isolates etc.  **Molecular epidemiology:** is the study of how genetic and environmental risk factors, identified at the **molecular** level, relate to epidemiological characteristics of the isolate (etiology, distribution and prevention of disease) | |  |
| **Sample Population** | | | | |  |
| What vector or host species were sampled in this article? (Including type of samples for humans)  *(Check all that apply)* | - **Humans**   **Samples taken to test for *Babesia sp.***:   - - Blood   - Lymph node   - Spleen   - CSF   - Other, please specify_____   - Questionnaire/focus group   **What best describes the human sample in this study?**   - “general population” - “at risk” population group: ____ - Only clinical Babesiosis cases are described in this paper. - hospital patient(s) - Patients attending doctors’ offices/ out-patient clinics - Sample library (stored isolates) - Yes, Other human population:____   **Note if co-infection or co-morbidity with *Babesia is presented for one or more patients in the results of this study***   - *Babesia sp.* co-infection - Borrelia burgdorferi (Lyme) - More than one Babesia sp. - Bartonella sp - Other specify: _____ - *Babesiosis*  co-morbidity - One or more Babesiosis patients had their spleen removed (or did not have a spleen) prior to infection.   **What was the most likely mode of human exposure for the cases reported in this paper?**   - Tick bite - Blood transfusion - Transplacental transmission - Direct contact with infected animals - Other: _____ - Not reported   **What type of diagnostic method was used to identify *Babesia sp.* infection in humans?**   - Molecular tests (e.g. RT-PCR)   - Immunoassay (e.g. ELISA, IFAT)   - Identification of parasite in blood (e.g. Blood smear and microscopic examination) - Other: _____ - N/A - **Other non-human host (reservoir) species sampled** *(dynamic list of hosts, add as we come across new ones.)*   Species part of sylvatic cycle, (note species): ______   - **Animal model experimental species sampled**: *(dynamic list of hosts, add as we come across new ones.)*   **What type of diagnostic method was used to identify *Babesia sp*. infection in animals?**   - Molecular tests (e.g. RT-PCR)   - Immunoassay(e.g. ELISA, IFAT)   - Identification of parasite in blood (e.g. Blood smear and microscopic examination) - Other: _____ - N/A - **Ticks** - *Ixodes cookie* - *Ixodes marxi* - *Ixodes pacificus* - *Ixodes persulcatus* - *Ixodes scapularis* - *Ixodes spinipalpus* - *Ixodes trianguliceps* - Ixodes *ricinus (Europe/Asia)* - *Dermacentor andersonii* - *Dermacentor variabilis* - *Dermacento reticulatus* - *Rhipicephalus sanguineus* - Add options as they occur   **What life stage of tick was sampled in this study:**   - Larvae - Nymph - Adult - Not reported.   **How were tick samples acquired?**   - From environment (e.g. drag sampling) - Removed from host, specify host(s): _________ - Other:______ - Laboratory population - Not reported - **Mosquitoes, specify species:** - Add options as they occur - **Other arthropod vector:** - Add options as they occur   **What type of diagnostic method was used to identify *Babesia sp.* infection in vectors?**   - Molecular tests (e.g. RT-PCR)   - Immunoassay(e.g. ELISA, IFAT)   - Identification of in sample (e.g. microscopic examination) - Other: _____ - N/A - **Parasite only studies using cell-cultures, in-vitro models** | | Population sampled for *Babesia sp.* refers to the human population. Please note classifying characteristics e.g. specific subset of the population and/or if co-infections or co-morbidities are reported.  **General population:** This sample could be representative of the general population e.g. blood donors, or random sample of people in an area etc.  **Clinical babesiosis** cases should be used if the report is a case report or series or a syndromic surveillance report as there is no control group and the catchment area is the hospital service area or the area represented by the syndromic surveillance.  Patients selected from **clinics, hospitals, laboratory submissions** etc. are not likely representative of the general population, but is representative of the people who go to a doctor for example.  **Other**, please indicate what the subset was. E.g. pregnant women, immunocompromised, hunters etc.  Select **No** if the sampling frame is an area, including all clinical cases in an area which we often see with case reports.  **Diagnostic test categories**  **Molecular diagnostic assays:**  Nested PCR, real-time PCR, reverse line blotting techniques. More sensitive (detection limit 50 or/ml) than blood smear and can id species, but is negative with treatment or in chronic animals  **Serology- immunoassays**: Indirect fluorescent antibody test (IFAT)- superior specificity, ELISA & dot-ELISA – superior sensitivity. Recombinant ELISAs have improved Sp. Species cross-react, thus not good to identify Babesia species and high sero-reactivity is common in endemic areas.  **Blood smear- microscopic examination**: definitive diagnosis= identification of parasites in stained erythrocytes (e.g. Giemsa, Diff-Quick, Romanowsky, Field’s or modified Wright’s stains). Light microscope detection limit is 0.001% parasitaemia= 5000 infected erythrocytes /mL. Low sensitivity, samples from peripheral capillaries like ear and nail bed work best.  **Sylvatic cycle:** Indicate if the author identifies species in the study as part of the sylvatic cycle.  **Animal Diagnostic Test Q:** Answer the diagnostic test question for both animal samples or animal models. | |  |
| **Pathogenesis- humans** | | | | |  |
| Are signs and symptoms of *Babesiosis* reported for the human sample population in this study? | - Yes - No | | Common symptoms: Fever, Chills, Sweats, Headache, Body ache, Anorexia, Nausea, Fatigue, Muscle pain, Non-productive cough (no mucus), Arthralgia (joint pain), Vomiting, ,Sore throat, Abdominal pain, Pink eye, Photophobia, Weight loss, Emotional lability, Depression, Hyperesthesis (more sensitive to stimuli),Pharyngeal erythema (redness in throat), Retinopathy (damage to retina), Retinal infacts, Hemolytic anemia, Jaundice, dark urine, unstable blood pressure, low platelet counts, disseminated intravascular coagulation (DIC), consumption coagulopathy, splenomegaly, hepatomegaly, Other vital organ malfunctions | |  |
| Are results of pathology or post-mortem investigations provided? | - Yes pathology - Yes post-mortem - No | | **Pathology** outcomes associated with the disease include any blood, urine, tissue or organ result using chemistry, clinical microbiology, hematology, molecular pathology and general pathology that characterises the effects of the disease on the body.  (Thus, initial biochemistry information or pathogen specific testing aimed at diagnosis does NOT constitute pathology unless there is a direct association with that outcome and disease pathology). | |  |
| Are sequelae reported following *Babesia sp.* infection in humans? | - Yes - No | |  | |  |
| Were risk factors for experiencing the above mentioned conditions reported by the author? | - Yes - No | |  | |  |
| Are the following characteristics of *Babesia* sp. infection in humans reported as an outcome of this study?  (Only answer if primary data is available) | - Time between exposure and becoming infectious (latent period) - Intrinsic incubation period (IIP) - Infectious period - Other: ___ | | **The intrinsic incubation period (IIP)** is the time between a human being becoming infected and the onset of symptoms due to the infection.  **Infectious period** is the time period in which humans could transmit the parasite. | |  |
| **Pathogenesis – all animals / animal models** | | | | |  |
| Are signs and symptoms of *Babesiosis* reported for the animal sample population in this study? | - Yes - No | |  | |  |
| Are results of pathology or post-mortem investigations provided? | - Yes pathology - Yes post-mortem - No | | **Pathology** outcomes associated with the disease include any blood, urine, tissue or organ result using chemistry, clinical microbiology, hematology, molecular pathology and general pathology that characterises the effects of the disease on the body.  (Thus, initial biochemistry information or pathogen specific testing aimed at diagnosis does NOT constitute pathology unless there is a direct association with that outcome and disease pathology). | |  |
| Are sequelae reported following *Babesia sp.* infection in animals? | - Yes - No | |  | |  |
| Are the following characteristics of *Babesia* sp. infection in animals reported as an outcome of this study?  (Only answer if primary data is available) | - Time between exposure and becoming infectious - Intrinsic incubation period (IIP) - Infectious period | | **The intrinsic incubation period (IIP)** is the time between a human being becoming infected and the onset of symptoms due to the infection.  **Infectious period** is the time period in which humans could transmit the parasite. | |  |
| **Treatment of Babesiosis** | | | | |  |
| What treatment options were examined for the treatment of *Babesia sp.* infections? | - Human treatments - Animal treatments - In vitro anti-parasitic experiment | | e.g. Plant-based inhibitors, non-steriodal anti-inflammatory drugs, corticosteroids, analgesics /anti-pyretic, anti-parasitic drugs, physical therapy or acupuncture, traditional medicine, etc. | |  |
| What drug or Other type of treatment was evaluated? | - Imidocarb dipropionate - Diminazene aceturate - Trypan blue - Atovaquone - Azithromycin - Diminazene - Clindamycin - Quinine - Oxomemaizine - Cotrimoxazole - Phenamindine - Pentamidine - Paraquone - Chloroquine - Tetracycline - Primaquine - Sulfadiazine - Pyrimethamine - Non-drug treatment (specify): _______ - *Add treatment drugs as required.* | | Current common treatments: Imidocarb dipropionate, Diminazene aceturate, Trypan blue, Atovaquoe, Azithromycin, Diminazene, Clindamycin  Older & discontinued treatments: Phenamindine, Pentamidine, Paraquone, Chloroquine  Shown not to work: Tetracycline,  Primaquine, Sulfadiazine, Pyrimethamine  **Non-drug treatments:** For any non-drug treatments, please ONLY use the one category and specify in the textbox what it is. E.g. erythrocyte exchange transfusion. | |  |
| Was the above noted treatment(s) evaluated for efficacy? | - Yes - No | | If there are multiple treatments and only some were evaluated for efficacy, choose Yes as there is “partial” data to extract. | |  |
| **Accuracy of Diagnostic Tests** | | | | |  |
| What tests were examined for their accuracy in the diagnosis of human cases and/or detection of *Babesia sp.*  in non-human hosts?  *(Please check all that apply)* | - Clinical diagnosis (by signs and symptoms) - Microscopic examination and staining - Serological Tests   - IFAT   - ELISA   - Dot-ELISA   - Other serological test, specify: ___ - Molecular Tests   - Nested PCR   - Real-time PCR   - Reverse line blotting techniques   - Other molecular tests, specify: __ - Other, specify:___ | | **Serological tests:** evaluate immune reaction to antigens.  **PCR:** Used to qualitatively detect gene expression through creation of complementary DNA transcripts from RNA | |  |
| Is information about sensitivity, specificity and/or raw data provided?  (Raw data should include 2x2 test agreement information and Sn/Sp data should include a measure of variability) | - Yes, sufficient extractable outcome data is provided. - No, insufficient data provided | | **Sensitivity** (also called the **true positive rate**) measures the proportion of positives that are correctly identified as such.  **Specificity** (also called the **true negative rate**) measures the proportion of negatives that are correctly identified as such. Eg. if 100 people known to have a disease were tested and 43 tested positive, the test has 43% sensitivity. If 100 people with no disease are tested and 96 return a negative result, then the test has **96%** specificity.  **Detection limits** – examples are cut off values for detecting positive or negative results for each test  If information about SN/SP/raw data/limitations of test, etc.. is provided for more than one test, select “Yes, for multiple tests” and more questions will become available. | |  |
| **Epidemiology Section**  *****ONLY put in populations where zoonotic babesia are found. If other babesia or no babesia were isolated from a population do NOT record it in this section.***** | | | | |  |
| **Describe the sample population(s) where the burden of infection for Babesiosis or Babesia sp. known to be zoonotic was reported in this study?** (Only answer if the data is a population sample.) | HUMANS  The sample represents [ date/ region/ population/n] = _____txt_______   - Sero-Prevalence - Case (disease) Prevalence - Incidence rate - Prevalence of Long-term sequelae - Case-fatality rate or proportion - Proportion hospitalized - Proportion asymptomatic - Other measure ____   HOSTS/RESERVOIRS  The sample represents [ date/ region/ population/n] = _____txt_______   - Sero-Prevalence - Case (disease) Prevalence - Incidence - Prevalence of Long-term sequelae - Case-fatality rate or proportion - Other measure ____   VECTORS  The sample represents [ date/ region/ population/n] = _____txt_______   - *Babesia sp.* infection prevalence - Babesia sp. infection Incidence - Other measure ____ | | **Sample:** describe what the sample represents – date, region, population sampled (n)  **Sero-prevalence:** Where a representative group of the target population is screened for sero-reactivity to *Babesia sp*.  **Case Prevalence:** It is the number of cases of babesiosis (individuals with infection) in a defined population at a specific point in time. Both numerator and denominator need to be provided.  **Incidence rate:** It is the number of new cases of babesiosis arising within a given time period in a specified population.  **Long-term sequelae:** Proportion of cases that develop chronic symptoms  **Case-fatality rate:** Proportion of cases that die from all the disease.  **Proportion** of cases that are **hospitalized**  **Other measures** e.g. prevalence of various co-infections or co-morbidities. | |  |
| If a Babesiosis **outbreak** is described in the paper, please indicate the total cases and location-year of the outbreak and whether fatalities and hospitalizations are reported.  (Only answer if this is an outbreak report) | The sample represents [ year/ region/ population]: ___  Outbreak cases; total number reported:____   - Case-fatality - hospitalizations | | Total includes sum of confirmed and probable.  **Confirmed cases** include all disease cases that are laboratory confirmed.  **Probable cases** are cases that are clinically diagnosed without laboratory confirmation | |  |
| If this report describes one or more **sporadic cases** of Babesiosis, indicate the # cases and year(s) and whether fatalities and hospitalizations are reported.  (Only answer if this is a sporadic cases report OR a case report/case series etc.) | The sample represents [ year/ region/ population]: ___  Sporadic cases; total number reported:____   - Case-fatality - hospitalizations | | **Sporadic cases** = When you see cases here and there. There is nothing linking one case to another. | |  |
| Were statistically significant risk factors for exposure to *Babesia sp.* reported in the study?  (Only applies to epidemiology studies: surveys, cross sectional, case control, cohort. **Not outbreak investigations**) | - Yes - No | |  | |  |
| **Transmission of *Babesia sp./*Parasite competence** | | | | |  |
| What aspect of transmission or parasite competence is examined in this paper?  (**List relevant outcomes ONLY** in the textboxes and note species if several species are examined in the paper). | - Vector to human - Human to vector - Vector to host - Host to vector - Vector to vector - Host to host - Human to human (e.g. intra-uterine) - Blood transfusion - Suitability of vector - Vector competence ___ - Vector behaviour ___ - Other vector attribute: _ - Other: _____ | | e.g. Time to transmission of pathogen. Vector life stage that transmission occurs, climatic factors (temp etc.) that impact transmission, etc.  **Vector competence** outcomes may include: lifespan/ life cycle, density, range of habitats, reproduction ranges, time required from vector infection until the pathogen reaches their salivary glands.  Vector behaviour: includes feeding behaviour, biting conditions, biting rate, etc. | |  |
| Surveillance | | | | |  |
| What type of surveillance program is described in the study?  *(Check all that apply)* | - Active: - Monitoring program - Passive   - Physician reporting - Laboratory-based - Event-based - Other, specify: _____ | | **Active surveillance**, in contrast to passive surveillance, requires that public health staff take direct action to collect disease information. For example, they may contact physicians, hospitals, laboratories, or other health entities to actively search for disease cases. Active surveillance may also occur through direct review of clinical or hospital charts, laboratory records, or emergency room patient logs. Active surveillance provides the most complete picture of disease incidence, i.e., cases are found in a timely manner, a greater number of cases are found, and more thorough information is obtained compared to passive surveillance methods. Active surveillance is an on-going activity and contains thresholds.  **Monitoring program:** Systematic purposeful program without active action plan. Simply counts numbers.  With **passive surveillance**, a member of the reporting community initiates a disease report that is communicated to a health department. For example, a physician may telephone a health department to discuss a case immediately upon seeing a patient with a suspected or confirmed case of a disease or an infection control practitioner may contact a health department upon receipt of positive laboratory results for a more common disease.  **Laboratory surveillance** differs from population-wide surveillance in that it can only monitor patients who are already receiving medical treatment and having lab tests done - does not identify patients who have never been tested.  **Event based surveillance** refers to the aggregation of data resulting from the monitoring of internet sources such as ProMed and GPHIN | |  |
| ***Babesia sp.* study – in vitro** | | | | |  |
| This study examines *Babesia sp.* with the following goal: | - Pathogenesis of *Babesia sp.* - *Babesia sp.* transmission characteristics - Molecular characterization of *Babesia sp.* - Phylogenetic tree - WGS sequencing | | Please indicate the objective/outcomes of the study under the appropriate category.  **Pathogenesis**: An *in vitro* parasite study on the pathogenesis of the parasite will look at factors that can up or down regulate / manage the parasite lifecycle to successfully cause infection/disease in the host. These include studies of immune reaction, production of antigens/antibodies in a cell culture.  **Transmission characteristics** are *in vitro* studies that examine, report on receptors, proteins etc. on or produced by the parasite that are necessary for *Babesia sp.* to move from one host to another.  **Molecular characterisation** studies in this group are *in vitro* studies examining the genetic make-up of the pathogen and identifying key areas of conservation or mutation. This may lead to a description of how changes in phenotype are related to genotype. Typically studies constructing phylogenetic trees to examine relatedness of isolates are part of this category. | |  |
| Is this a molecular epidemiology study?  (*Appears here and under the epidemiology section)* | - Yes - No | | **Molecular epidemiology** is a branch of **epidemiology** and medical science that focuses on the contribution of potential genetic and environmental risk factors, identified at the **molecular** level, to the etiology, distribution and prevention of disease within families and across populations | |  |
| **Intervention/ Mitigation Strategies** | | | | |  |
| What prevention/control strategies were investigated?  (Please check all that apply) | | - Human behavioural protective measures: - Wearing long pants and/or lightly-coloured clothing - Tucking pants into socks - Using repellents - Wearing clothing treated with permethrin insecticide - Using bed nets - Having window/door screens - Emptying standing water from containers such as flowerpots or buckets and cleaning them (mosquito intervention) - Avoiding tick infested areas - Other behavioural measure, _____ - Intervention for blood supply - Vaccination - Landscape modification - Chemical control measures: - Insecticides - Other chemical control measures:____ - Biologic control of vector - Public education - Other mitigation/intervention:___ | | **PPMs**  Are behaviours that may reduce the risk of contact with a tick or exposure to *Babesia sp.* or development of disease from *Babesia sp*. This may include protective clothing, repellents, barriers between the home and outside, destruction of tick habitat close to home.  Other interventions may include:   - Protection of the blood supply - Vaccination (none are known) - Landscape modification (frequent mowing, branch trimming, leaf litter clearing, removal of bird feeders, fencing to keep deer out, and use of mulch or gravel as a dry barrier between lawn and woods. - biological control (exposing ticks to *H. hookeri*, fungi, nematodes and viruses) - Public education to decrease the risk of tick contact for humans and companion animals. | |
| Did the authors describe the impact of the mitigation/intervention strategy? | - Yes - No | | Briefly highlight the findings of the evaluation under successes and limitations. | |  |
| **Social Impact** | | | | |  |
| Did the paper investigate knowledge and attitudes and/or risk perceptions towards Babesiosis and/or *Babesia sp.* and potential prevention and control strategies?  (please check all that apply) | - Yes, **concerns** about toxic or environmental effects of control measures (e.g. DEET) - Yes, **perceptions** about the severity of Babesiosis or vulnerabilities - Yes, **perceived** efficacy of protective measures - Yes, **knowledge** on behavioural mitigation practices - Yes, **knowledge** on Babesiosis disease/ Babesia sp. infection - Yes, **knowledge** on *Babesia sp.* harbouring vectors - Yes, **public attitudes** towards paying for protection from *Babesia sp.* (willingness to pay) - Yes, other:______ | |  | |  |
| Does this paper focus on Babesiosis and/or *Babesia sp.*? | - Yes, the data in this paper is specific to Babesiosis and/or *Babesia sp.* - No, this paper more broadly refers to tickborne (or other vectorborne) diseases. | |  | |  |
| What specific populations were investigated for contextual information?  *(please check all that apply)* | - General public - Physicians - Other medical or public health professionals - Government personnel - NGO personnel - Other, please specify _____ | | What populations did the researchers speak to? Gather information from? | |  |
| Economic Burden | | | | |  |
| Does the article report on the economic burden of Babesiosis and/or *Babesia sp.* or cost-benefit of control measures?  (Check all that apply) | - Yes, economic burden - Yes, cost-benefit of control measures - Yes, Other economic measure - No data is reported in the paper although economics is discussed. | |  | |  |
| Predictive Model for Babesiosis and/or *Babesia sp.* | | | | |  |
| Does this predictive model include Canada or at least the USA? | - Yes, Canada - Yes, USA but not Canada - No | |  | |  |
| Does the model include predictions under different climate change scenarios, to explore the impact of spread or emergence of Babesiosis and/or *Babesia sp.* in the future? | - Yes, climate change scenarios are explored - No, the model is built upon current climate parameters. - No, other. Explain___ | |  | |  |
| **Other Babesiosis and/or *Babesia sp.* topics** | | | | |  |
| Describe the Babesiosis and/or *Babesia sp.* topic discussed in the research article that doesn't fit into the previous categories. | ___TXT__ | |  | |  |
| **Final Section** | | | | |  |
| Does the article investigate or discuss the potential impacts of climate change (CC) on Babesiosis or *Babesia sp.*? | - Yes, CC discussed - No | | If the impacts of climate change are investigated as part of the study or are discussed in the discussion based upon the research findings, check yes to this question. | |  |
| Is there sufficient quantitative extractable data in this paper to proceed to quality assessment and further data extraction? | - Yes, one or more outcomes can be extracted and potentially used for meta-analysis. - No, data is not quantitative (e.g. phylogenetic trees) - No, study is not reported well enough to extract any quantitative outcomes or data is presented only in graphs. | | Quick QA on whether study is worth progressing to QA/DE levels and more in depth analysis. Are the outcomes reported in a manner that we can extract them?  Yes= at least 1 outcome has sufficient extractable data.  No, not quantitative: case studies, phylogenetic trees.  No, not reported sufficiently – there is not a single quantitative outcome that could be extract and used for meta-analysis- = can’t extract a numerator and denominator, missing summary measures and/or measure of variability, no sample size, all in graphs | |  |
| Additional comments: Are there any important details that you believe were not extracted? | __TXT___ | |  | |  |

Articles will be double extracted in Distiller SR.

## Appendix 3: Search Strategy Implemented

**Library/Database:** PubMed 
**Date of Search:** February 20, 2017
**Search String:** All fields (babesiosis and (human or humans or man or woman or people)) OR babesia
**# Hits:** 5042

**Library/Database:** Scopus
**Date of Search:** February 20, 2017
**Search String:** Article Title, Abstract, Keywords (babesiosis and (human or humans or man or woman or people)) OR babesia
**# Hits:** 6276

**Library/Database:** Cochrane Library
**Date of Search:** February 20, 2017
**Search String:** Title, Abstract, Keyword (babesiosis and (human or humans or man or woman or people)) OR babesia
**# Hits:** 7

**Library/Database:** Global Health /EMBASE 1974-, searched together as they are provided by OVID via the PHAC library.
**Date of Search:** February 20, 2017
**Search String:** Title, Abstract, Keyword, Original Title (babesiosis and (human or humans or man or woman or people)) OR babesia
**# Hits:** 7108

**# Hits:**
Total # of citations prior to de-duplication: 18433

**Updated Search (Filter articles 2017-2018)**

**Library/Database:** PubMed

**Date of Search:** May 10, 2018

**Search String**: All fields (babesiosis and (human or humans or man or woman or people)) OR babesia

**# Hits:** 420

**Library/Database:** Scopus

**Date of Search:** May 10, 2018

**Search String:** Article Title, Abstract, Keywords (babesiosis and (human or humans or man or woman or people)) OR babesia

**# Hits:** 472

**Library/Database:** Cochrane Library

**Date of Search:** May 10, 2018

**Search String:** Title, Abstract, Keyword (babesiosis and (human or humans or man or woman or people)) OR babesia

**# Hits:** 2

**Library/Database:** Global Health /EMBASE 1974-, searched together as they are provided by OVID via the PHAC library.

**Date of Search:** May 10, 2018

**Search String:** Title, Abstract, Keyword, Original Title (babesiosis and (human or humans or man or woman or people)) OR babesia

**# Hits:** 570

**# Hits (updated search):**

Total # of citations prior to de-duplication: 1464

Total # citations added from the grey literature search = 22

Total # of citations added from search validation = 6

**Initial and updated search combined:**

Total # of citations prior to de-duplication: 19925

Total # of citations after round of de-duplication: 7538

**Grey Literature Search Hits Breakdown**

Provincial/State Google Search Hits

Canada 1

USA 10

Ministry of Health/Public Health Google Search

CDC 3

Datacite Search 3

Europe, Asia, and South America Google Search 5

**Websites searched for Grey Literature**

**Africa**

http://www.mohp.gov.eg/

<http://www.health.go.ke/?s=Babesia>

<http://www.sante.gov.mg/home/n>

<http://www.health.gov.ng/index.php/component/search/?searchword=Babesia&searchphrase=all&Itemid=435>

<http://www.health.gov.za/index.php?searchword=Babesia&option=com_search&searchphrase=all&Itemid=237>

<http://www.santetunisie.rns.tn/fr/recherche?searchword=Babesia&searchphrase=all>

<https://healthresearchweb.org/en/zimbabwe/institution_201>

**Australia & New Zealand**

<http://www.health.gov.au/>

<https://www.health.govt.nz/>

**Central & South America**

<https://www.argentina.gob.ar/buscar/Babesia>

<https://www.minsalud.gov.co/sites/english/Pages/default.aspx>

<http://www.dominicanrepublic.com/?s=Babesia&submit>=

<http://www.gov.gd/search_result.html?cx=004548860350108938520%3Ayid_mrmtt4o&cof=FORID%3A10&ie=UTF-8&q=Babesia&x=0&y=0>

<https://www.gob.mx/salud/en>

<http://moh.gov.ms/?s=Babesia>

**Asia**

<https://www2.chp.gov.hk/search/en/chp_search_result.php?q=Babesia>

<https://phfi.org/>

<https://www.health.gov.il/English/MinistryUnits/HealthDivision/PublicHealth/Pages/Default.aspx>

<https://www.niph.go.jp/index_en.html>

<http://www.mohw.go.kr/eng/cs/scs0501mn.jsp?PAR_MENU_ID=1004&MENU_ID=100409#AtoZ_B>

<http://nih.org.pk/?page_id=629>

**Europe**

https://www.antsz.hu/en/about_us

http://www.insp.gov.ro/

https://phc.org.ua/en

http://government.ru/en/department/23/events/

http://sid.usal.es/centrosyservicios/discapacidad/291/2-1-1-1/insalud-instituto-nacional-de-la-salud.aspx

https://eupha.org/german-public-health-association-dgph

http://invs.santepubliquefrance.fr/en

http://www.salute.gov.it/portale/salute/p1_4.jsp?area=Il_Ssn

https://eupha.org/austrian-public-health-association#

https://www.health.belgium.be/en/search?keyword=Babesia

http://minzdrav.gov.by/en/

http://www.mh.government.bg/en/ministry/secondary-authorizing-officers/national-center-public-health-and-analysis/

https://www.hzjz.hr/en/

http://www.szu.cz/index.php?lang=2

http://www.si-folkesundhed.dk/?lang=en

http://www.tai.ee/en/about-us/national-institute-for-health-development

https://www.gtp.gr/TDirectoryDetails.asp?ID=12324

https://www.landlaeknir.is/english/

http://www.vm.gov.lv/en/

https://sam.lrv.lt/en/health-care/public-health

http://www.innovation.public.lu/en/cooperer/trouver-partenaires/instituts/lih1/lih1/department-of-public-health/index.html

http://msmps.gov.md/en

http://www.mzdravlja.gov.me/en/search?query=Babesia&sortDirection=desc

https://www.rivm.nl/en/Search?searchbase=0&searchrange=10&searchpage=1&freetext=Babesia&submit=Search

https://www.fhi.no/en/el/insects-and-pests/ticks-and-tick-borne-diseases/other/

http://www.mz.gov.pl/en/

https://www.dgs.pt/directorate-general-of-health/about-us.aspx

http://www.zdravlje.gov.rs/

http://www.uvzsr.sk/en/

https://www.folkhalsomyndigheten.se/the-public-health-agency-of-sweden/search/?q=Babesia

https://www.swisstph.ch/en/about/eph/

https://www.saglik.gov.tr/arama?_Dil=2

https://www.gov.uk/search?q=Babesia&show_organisations_filter=true

**United States of America**

https://www.cdc.gov/mmwr/volumes/64/wr/mm6453a1.htm

https://www.health.ny.gov/statistics/diseases/communicable/2016/docs/cases.pdf

http://www.mass.gov/eohhs/docs/dph/cdc/babesios-surveillance-2015.pdf

http://www.nj.gov/health/cd/documents/reportable_disease_magnet.pdf

https://www.dhs.wisconsin.gov/disease/babesiosisdata.htm

http://www.health.state.mn.us/divs/idepc/newsletters/dcn/sum16/2016dcn.pdf

http://www.portal.ct.gov/DPH/Infectious-Diseases/ID-Home/Infectious-Diseases-Statistics#47477

http://www.ri.gov/press/view/6948; <http://www.rimed.org/rimedicaljournal/2014/09/2014-09-46-health-lawrence.pdf>

<http://www.ct.gov/caes/lib/caes/documents/publications/press_releases/2016/increased_prevalence_of_babesiosis_and_lyme_disease_in_ct.pdf>

<https://www.health.ny.gov/press/releases/2017/2017-08-29_tick_outreach.htm>

https://www.tickreport.com/

http://www.nj.gov/health/cd/documents/reportable_disease/2016webstatistics.pdf

https://www.cdc.gov/mmwr/volumes/66/wr/mm6626a2.htm

http://www.health.state.mn.us/divs/idepc/newsletters/dcn/sum16/babesiosis.html

http://www.rimed.org/rimedicaljournal/2017/11/2017-11-41-health-barkley.pdf

**Canada**

http://www.pwsd76.ab.ca/Resources/policies/Documents/IHCF_Ex2NotifiableDiseaseList.pdf

http://www.bccdc.ca/resource-gallery/Documents/Statistics%20and%20Research/Statistics%20and%20Reports/Epid/Annual%20Reports/Reportable%20Disease%20Summary%20Tables.pdf

https://www.gov.mb.ca/health/publichealth/cdc/tickborne/index.html

http://laws.gnb.ca/en/showfulldoc/cr/2009-136//2013110maladies_transmissibles.html

http://www.health.gov.nl.ca/health/publichealth/cdc/notifiable_disease_list.pdf

https://novascotia.ca/dhw/CDPC/documents/2017_Tick_Borne_Disease_Response_Plan.pdf, have copy; no cases reported

https://www.princeedwardisland.ca/en/information/health-and-wellness/pei-communicable-disease-guidelines

http://www.msss.gouv.qc.ca/professionnels/maladies-a-declaration-obligatoire/mado/demarche-pour-les-medecins/

https://www.publichealthontario.ca/en/eRepository/Reportable_Diseases_CDPC_Contact_List.pdf

https://www.saskatchewan.ca/government/government-structure/ministries/health/other-reports/public-health-monitoring-and-surveillance

http://www.yhssa.hss.gov.nt.ca/sites/default/files/nwt_communicable_disease_report_form.pdf

<http://www.hss.gov.yk.ca/pdf/comm_diseases.pdf>

https://www1.agric.gov.ab.ca/$department/deptdocs.nsf/all/cpv12455

https://www2.gov.bc.ca/gov/content/industry/agriculture-seafood/animals-and-crops/animal-health/reportable-notifiable-diseases/reportable-diseases

https://web2.gov.mb.ca/laws/regs/current/_pdf-regs.php?reg=37/2009

http://www2.gnb.ca/content/dam/gnb/Departments/h-s/pdf/en/CDC/HealthProfessionals/Annual_Report_CDC_Branch_2016.pdf

http://www.assembly.nl.ca/Legislation/sr/Regulations/rc120037.htm

http://www.cbc.ca/news/canada/prince-edward-island/pei-provincial-veterinarian-1.4152048

https://www.saskatchewan.ca/business/agriculture-natural-resources-and-industry/agribusiness-farmers-and-ranchers/livestock/animal-health-and-welfare/notifiable-disease-list

https://www.alberta.ca/release.cfm?xID=469484F08673C-BD66-2FD2-493E95149CA64631

http://www.wallisroughley.ca/ticksurveillance.html

http://www2.gnb.ca/content/gnb/en/departments/ocmoh/cdc/content/vectorborne_andzoonotic/lyme/tick_testing.html

http://www.faa.gov.nl.ca/agrifoods/animals/health/pdf/tick_submission.pdf

http://www.gov.pe.ca/photos/original/hpei_lymediseas.pdf

https://www.inspq.qc.ca/sites/default/files/publications/2165_surveillance_lyme_diseases_ixodes_scapularis.pdf

https://www.publichealthontario.ca/en/eRepository/PHU_Tick_surveillance_quick_guide.pdf

http://sdcl-testviewer.ehealthsask.ca/SCI/Requisitions/Tick%20Submission%20Form.pdf; fprwarded nml for lyme and species id

https://www.canadianveterinarians.net/provincial-tick-resources
