# Supplementary material for: Zoonotic Babesia: A scoping review of the global evidence
Source: PLoS One. 2019 Dec 30;14(12):e0226781. doi: 10.1371/journal.pone.0226781 (PMC6936817; doi:10.1371/journal.pone.0226781)
Supplement: S1 Table — (DOCX) [file pone.0226781.s004.docx]

**S1 Table.** Host to vector and vector to host *Babesia* transmission scenarios

| Animal species* | Tick species | *Babesia* Species | Reference |
| --- | --- | --- | --- |
| Host to Vector transmission (n=21) | | | |
| Balb/c mice | *Rhipicephalus haemaphysaloides* | *B. microti* | (Li et al. 2016) |
| CB17 SCID mice | *Rhipicephalus haemaphysaloides* | *B. microti* | (Li et al. 2016) |
| CBA/C57 mice | *Ixodes trianguliceps* | *B. microti* | (Randolph 1991) |
| ICR mice | *Haemaphysalis longicornis* | *B. microti* | (Kusakisako et al. 2015) |
| White-footed mice | *Ixodes scapularis* | *B. microti* | (Piesman and Spielman 1982, Mather et al. 1990, Telford and Spielman 1993, Dunn et al. 2014) |
| Bank voles | *Ixodes trianguliceps* | *B. microti* | (Hussein 1980, Randolph 1991, Randolph 1995) |
| Hamsters^a^ | *Ixodes scapularis* | *B. microti* | (Piesman and Spielman 1980, Piesman and Spielman 1982, Rudzinska et al. 1983, Piesman 1988) |
| Golden Syrian hamsters | *Ixodes scapularis* | *B. microti* | (Spielman 1976, Oliveira 1979) |
|  | *Dermacentor andersonii* | *B. microti* | (Genga and Kreier 1976, Oliveira 1979) |
|  | *Demicentor variabilis* | *B. microti* | (Oliveira 1979) |
|  | *Ixodes pacificus* | *B. microti* | (Oliveira 1979) |
| Gerbils^a^ | *Dermacentor andersonii* | *B. microti* | (Oliveira 1979) |
|  | *Demicentor variabilis* | *B. microti* | (Oliveira 1979) |
|  | *Ixodes pacificus* | *B. microti* | (Oliveira 1979) |
|  | *Ixodes scapularis* | *B. microti* | (Oliveira 1979) |
|  | *Ixodes ricinus* | *B. microti* | (Gray et al. 2002, Mazyad et al. 2010) |
|  | *Ixodes ricinus* | *B. divergens* | (Lewis and Young 1980) |
| Cattle | *Ixodes ricinus* | *B. divergens* | (Joyner et al. 1963, Donnelly and Peirce 1975, Lewis and Young 1980) |
| New Zealand white rabbits | *Rhipicephalus appendiculatus* | *B. microti* | (Irvin and Brocklesby 1972) |
| Vector to Host Transmission (n=22) | | | |
| Balb/c mice | *Rhipicephalus haemaphysaloides* | *B. microti* | (Li et al. 2016) |
| CB17 SCID mice | *Rhipicephalus haemaphysaloides* | *B. microti* | (Li et al. 2016) |
| ICR mice | *Haemaphysalis longicornis* | *B. microti* | (Kusakisako et al. 2015) |
| White-footed mice | *Ixodes scapularis* | *B. microti* | (Mather et al. 1990) |
| Kunming mice | *Haemaphysalis longicornis* | *B. microti* | (Wu et al. 2017) |
| Prairie voles | *Ixodes spinipalpus* | *B. microti* | (Burkot et al. 2000) |
| Bank voles | *Ixodes trianguliceps* | *B. microti* | (Hussein 1980, Randolph 1995) |
|  | *Ixodes canisuga* | *B. microti* | (Hussein 1980) |
| Hamsters^a^ | *Ixodes scapularis* | *B. microti* | (Piesman and Spielman 1980) |
| Golden Syrian hamsters | *Ixodes persulcatus* | *B. microti* | (Zamoto-Niikura et al. 2012, Zamoto-Niikura et al. 2016) |
|  | *Ixodes ovatus* | *B. microti* | (Zamoto-Niikura et al. 2012) |
|  | *Ixodes pacificus* | *B. microti* | (Oliveira and Kreier 1979, Oliveira 1979) |
|  | *Ixodes scapularis* | *B. microti* | (Spielman 1976, Oliveira and Kreier 1979, Oliveira 1979, Piesman et al. 1987) |
|  | *Dermacentor andersonii* | *B. microti* | (Genga and Kreier 1976, Oliveira and Kreier 1979, Oliveira 1979) |
|  | *Dermacentor variabilis* | *B. microti* | (Oliveira and Kreier 1979, Oliveira 1979) |
| Gerbils^a^ | *Ixodes ricinus* | *B. microti* | (Gray et al. 2002, Mazyad et al. 2010) |
|  | *Ixodes pacificus* | *B. microti* | (Oliveira and Kreier 1979, Oliveira 1979) |
|  | *Ixodes scapularis* | *B. microti* | (Oliveira and Kreier 1979, Oliveira 1979) |
|  | *Dermacentor andersonii* | *B. microti* | (Oliveira and Kreier 1979, Oliveira 1979) |
|  | *Dermacentor variabilis* | *B. microti* | (Oliveira and Kreier 1979, Oliveira 1979) |
|  | *Ixodes ricinus* | *B. divergens* | (Lewis and Young 1980) |
| Mongolian gerbils | *Ixodes persulcatus* | *B. microti* | (Zamoto-Niikura et al. 2016) |
| Cattle | *Ixodes ricinus* | *B. divergens* | (Joyner et al. 1963, Donnelly and Peirce 1975, Lewis and Young 1980) |
| New Zealand white rabbits | *Rhipicephalus appendiculatus* | *B. microti* | (Irvin and Brocklesby 1972) |
| Rhesus macaques | *Ixodes scapularis* | *B. microti* | (Ruebush Ii et al. 1981) |

^a^ Species not specified.

* Transmission scenario successes/failures were not captured in this ScR.
