# Supplementary material for: Zoonotic Babesia: A scoping review of the global evidence
Source: PLoS One. 2019 Dec 30;14(12):e0226781. doi: 10.1371/journal.pone.0226781 (PMC6936817; doi:10.1371/journal.pone.0226781)
Supplement: S2 Table — (DOCX) [file pone.0226781.s005.docx]

**S5 Table.** Details of animal epidemiology outcomes and sporadic case reports by country and animal species. All values are counts of individual articles.

| **Population Category** | **Animal Host Species (N)** | ***Babesia Sp.* reported** | **Sero-prevalence** | **Case-prevalence** | **Outbreak** | **Sporadic cases** |
| --- | --- | --- | --- | --- | --- | --- |
| **North America** | | | | | | |
| **United States (37)** | | | | | | |
| Rodents (29) | White-footed mice (20) | *B. microti* | 8 | 12 |  |  |
|  | Deer mice (1) | *B. microti* |  | 1 |  |  |
|  | California mice (1) | *B. microti* |  | 1 |  |  |
|  | Mice^a^ (1) | *B. microti* |  | 1 |  |  |
|  | Cotton rats (1) | *B. microti* |  | 1 |  |  |
|  | Brown rats (1) | *B. microti* |  | 1 |  |  |
|  | Desert woodrats (1) | *B. microti* |  | 1 |  |  |
|  | Prairie voles (2) | *B. microti* |  | 2 |  |  |
|  | Montane voles (1) | *B. microti* |  | 1 |  |  |
|  | Meadow voles (7) | *B. microti* | 1 | 7 |  |  |
|  | Water voles (1) | *B. microti* |  | 1 |  |  |
|  | Southern red-backed voles (1) | *B. microti* |  | 1 |  |  |
|  | Northern red-backed voles (1) | *B. microti* |  | 1 |  |  |
|  | Tundra voles (1) | *B. microti* |  | 1 |  |  |
|  | Singing voles (1) | *B. microti* |  | 1 |  |  |
|  | California voles (1) | *B. microti* |  | 1 |  |  |
|  | Unknown vole sp. (1) | *B. microti* |  | 1 |  |  |
|  | Chipmunks (2) | *B. microti* |  | 2 |  |  |
|  | California Ground Squirrel (1) | *B. microti* |  | 1 |  |  |
| Sorex (3) | Short-tailed shrew (2) | *B. microti* |  | 2 |  |  |
|  | Masked shrew (1) | *B. microti* |  | 1 |  |  |
|  | Shrews^a^ (1) | *B. microti* |  | 1 |  |  |
| Cervids (1) | White-tailed deer (1) | *B. microti* |  | 1 |  |  |
| Leporids (4) | Eastern cottontails (3) | *B. microti* (2), *B. divergens* (1), *B. sp. MO1* (1) | 2 | 3 |  |  |
|  | Desert cottontails (1) | *B. microti* |  | 1 |  |  |
| Ursids (2) | American black bears (2) | *B. microti* |  | 2 |  |  |
| Monkeys (1) | Wild baboons (1) | *B. microti* |  |  |  | 1 |
| Others (1) | North American racoons (1) | *B. microti* |  | 1 |  |  |
| **Europe** | | | | | | |
| **United Kingdom (Great Britain, Ireland, Scotland) (20)** | | | | | | |
| Rodents (9) | Yellow-necked mice (1) | *B. microti* | 1 |  |  |  |
|  | Wood mice (3) | *B. microti* | 2 | 1 |  |  |
|  | Field voles (6) | *B. microti* |  | 5 |  |  |
|  | Bank voles (4) | *B. microti* | 2 | 2 |  |  |
|  | Water voles (1) | *B. microti* |  | 1 |  |  |
| Bovids (7) | Dairy cattle (1) | *B. divergens* | 1 |  |  |  |
|  | Beef cattle (1) | *B. divergens* | 1 |  |  |  |
|  | Cattle^a^ (6) | *B. divergens* | 6 |  | 1 |  |
| Cervids (4) | Reindeer (1) | *B. divergens* |  |  | 1 |  |
|  | Red deer (3) | *B. divergens* | 2 | 1 |  |  |
|  | Sika deer (1) | *B. divergens* | 1 |  |  |  |
| Sorex (3) | Common shrews (3) | *B. microti* | 1 | 2 |  |  |
|  | Eurasian pygmy shrews (1) | *B. microti* | 1 |  |  |  |
|  | Eurasian water shrews (1) | *B. microti* | 1 |  |  |  |
| Other (1) | Least weasel (1) | *B. microti* | 1 |  |  |  |
|  | European moles (1) | *B. microti* |  | 1 |  |  |
| **France (17)** | | | | | | |
| Rodents (1) | Bank voles (1) | *B. microti* |  | 1 |  |  |
| Bovids (14) | Dairy cows (2) | *B. divergens* | 2 |  |  |  |
|  | Cattle^a^ (12) | *B. divergens* | 6 | 6 | 1 | 3 |
| Cervids (2) | Roe deer (2) | *B. divergens* (1), *B. venatorum* (2) | 1 | 2 |  |  |
| **Poland (14)** | | | | | | |
| Rodents (11) | Yellow-necked mice (4) | *B. microti* |  | 4 |  |  |
|  | Striped-field mice (2) | *B. microti* |  | 2 |  |  |
|  | Common voles (3) | *B. microti* |  | 3 |  |  |
|  | Tundra voles (4) | *B. microti* |  | 4 |  |  |
|  | Bank voles (6) | *B. microti* |  | 5 |  | 1 |
|  | Field voles (1) | *B. microti* |  | 1 |  |  |
| Cervids (2) | Red deer (1) | *B. divergens* |  | 1 |  |  |
|  | Roe deer (2) | *B. venatorum* (1), *B. divergens* (2) |  | 2 |  |  |
| Canids (1) | Red foxes (1) | *B. microti* |  | 1 |  |  |
| **Italy (13)** | | | | | | |
| Bovids (5) | Dairy cattle (1) | *B. divergens* |  | 1 |  |  |
|  | Cattle^a^ (3) | *B. microti* (1), *B. microti-like* (1), *B. divergens* (3) | 2 | 3 |  |  |
|  | Chamois (1) | *B. divergens* |  | 1 |  |  |
| Cervids (4) | Red deer (1) | *B. divergens* |  | 1 |  |  |
|  | Roe deer (3) | *B. divergens* (2), *B. divergens-like* (1), *B. microti-like* (1), *B.* *venatorum* (2), *B. sp. MO1* (1) |  | 3 |  | 1 |
| Canids (3) | Domestis dogs^a^ (1) | *B. microti* | 1 | 1 |  |  |
|  | Red foxes (2) | *B. microti-like* |  | 2 |  |  |
| Felines (3) | Domestic Cats^a^ (3) | *B. microti* | 2 | 2 |  |  |
| Other (1) | Horses^a^ (1) | *B. microti-like* |  | 1 |  |  |
|  | Ungulates^a^ (1) | *B. microti-like, B. divergens-like, B. venatorum* |  | 1 |  |  |
| **Spain (9)** | | | | | | |
| Cervids (1) | Roe deer (1) | *B. divergens* |  | 1 |  |  |
| Bovids (4) | Friesian cows (1) | *B. divergens* |  | 1 |  |  |
|  | Cattle^a^ (3) | *B. divergens* |  | 3 |  |  |
| Canids (4) | Domestic dogs^a^ (4) | *B. microti-like* |  | 2 |  | 2 |
| **Germany (8)** | | | | | | |
| Rodents (4) | Yellow-necked mice (1) | *B. microti* |  | 1 |  |  |
|  | Striped field mice (1) | *B. microti* |  | 1 |  |  |
|  | Common voles (3) | *B. microti* |  | 3 |  |  |
|  | Bank voles (3) | *B. microti* |  | 3 |  |  |
|  | Field voles (1) | *B. microti* |  | 1 |  |  |
| Cervids (3) | Roe deer (2) | *B. venatorum* |  | 2 |  |  |
|  | Fallow deer (1) | *B. venatorum* |  | 1 |  |  |
|  | Reindeer (1) | *B. divergens, B. venatorum* |  | 1 |  |  |
| Bovids (1) | Mouflons (1) | *B. venatorum* |  | 1 |  |  |
| Canids (1) | Dogs^a^ (1) | *B. microti-like* |  |  |  | 1 |
| Other (1) | Common mole (1) | *B. venatorum* |  | 1 |  |  |
| **Switzerland (7)** | | | | | | |
| Rodents (1) | Field voles (1) | *B. microti* | 1 | 1 |  |  |
|  | Bank voles (1) | *B. microti* | 1 | 1 |  |  |
| Bovids (6) | Cattle^a^ (4) | *B. divergens* | 1 | 2 |  | 1 |
|  | Chamois (2) | *B. venatorum* (1), *B. divergens-like* (1) |  | 1 |  | 1 |
|  | Ibex (1) | *B. venatorum* |  | 1 |  |  |
| Cervids (1) | Roe deer (1) | *B. venatorum* |  | 1 |  |  |
|  | Red deer (1) | *B. divergens* |  | 1 |  |  |
| **Croatia (3)** | | | | | | |
| Rodents (3) | Yellow-necked mice (3) | *B. microti* |  | 3 |  |  |
|  | Striped field mice (2) | *B. microti* |  | 2 |  |  |
|  | Wood mice | *B. microti* |  | 1 |  |  |
|  | Field voles (1) | *B. microti* |  | 1 |  |  |
|  | Bank voles (2) | *B. microti* |  | 2 |  |  |
| **Norway (3)** | | | | | | |
| Rodents (1) | Wood mice (1) | *B. microti* |  | 1 |  |  |
|  | Bank voles (1) | *B. microti* |  | 1 |  |  |
|  | Field voles (1) | *B. microti* |  | 1 |  |  |
| Bovids (2) | Cattle^a^ (2) | *B. divergens* | 1 |  |  | 1 |
| **Portugal (3)** | | | | | | |
| Bovids (3) | Cattle^a^ (3) | *B. divergens* |  | 3 |  |  |
| **Austria (2)** | | | | | | |
| Bovids (1) | Cattle^a^ () | *B. divergens* |  |  |  | 1 |
| Cervids (1) | Red deer (1) | *B. divergens* |  | 1 |  |  |
| **Belgium (2)** | | | | | | |
| Bovids (1) | Cattle^a^ (1) | *B. divergens* | 1 |  |  |  |
| Canids (1) | Brittany spaniel dogs (1) | *B. microti* |  |  |  | 1 |
| **Netherlands (2)** | | | | | | |
| Bovids (1) | Cattle^a^ (1) | *B. divergens* | 1 |  |  |  |
| Cervids (1) | Reindeer (1) | *B. venatorum* |  |  |  | 1 |
| **Slovakia (2)** | | | | | | |
| Rodents (2) | Yellow-necked mice (2) | *B. microti* |  | 2 |  |  |
|  | Striped-field mice (1) | *B. microti* |  | 1 |  |  |
|  | Common voles (1) | *B. microti* |  | 1 |  |  |
|  | Bank voles (2) | *B. microti* |  | 2 |  |  |
| **Slovenia (2)** | | | | | | |
| Rodents (1) | Yellow-necked mice (1) | *B. microti* |  | 1 |  |  |
|  | Bank voles (2) | *B. microti* |  | 1 |  |  |
| Cervids (1) | Red deer (1) | *B. divergens, B. venatorum* |  | 1 |  |  |
|  | Roe deer (1) | *B. divergens, B. venatorum* |  | 1 |  |  |
| **Sweden (2)** | | | | | | |
| Bovids (1) | Cattle^a^ (1) | *B. divergens* |  | 1 |  |  |
| Cervids (1) | Roe deer (1) | *B. venatorum* |  | 1 |  |  |
| **Bulgaria (1)** | | | | | | |
| Rodents (1) | Wood mice (1) | *B. microti* |  | 1 |  |  |
| **Czech Republic (1)** | | | | | | |
| Rodents (1) | Bank voles (1) | *B. microti* |  | 1 |  |  |
|  | Common voles (1) | *B. microti* |  | 1 |  |  |
|  | Field voles (1) | *B. microti* |  | 1 |  |  |
| **Finland (1)** | | | | | | |
| Rodents (1) | Bank voles (1) | *B. microti* |  | 1 |  |  |
| **Greece (1)** | | | | | | |
| Bovids (1) | Cattle^a^ (1) | *B. divergens* | 1 |  |  |  |
|  | Goats^a^ (1) | *B. divergens* | 1 |  |  |  |
|  | Sheep^a^ (1) | *B. divergens* | 1 |  |  |  |
| **Hungary (1)** | | | | | | |
| Bovids (1) | Cattle^a^ (1) | *B. divergens* |  | 1 |  |  |
| **Serbia (1)** | | | | | | |
| Canids (1) | Domestis dogs^a^ (1) | *B. microti* |  | 1 |  |  |
| **Yugoslavia (1)** | | | | | | |
| Rodents (1) | Yellow-necked mice (1) | *B. microti* |  | 1 |  |  |
|  | Striped field mice (1) | *B. microti* |  | 1 |  |  |
|  | House mice (1) | *B. microti* |  | 1 |  |  |
| Sorex (1) | Mediterranean water shrew (1) | *B. microti* |  | 1 |  |  |
| **Asia** | | | | | | |
| **Japan (11)** | | | | | | |
| Rodents (7) | Japanese field mice (7) | *B. microti* (4), *B. microti-like* (2) | 1 | 7 |  |  |
|  | Anderson's red backed voles (1) | *B. microti* |  | 1 |  |  |
|  | Grey red-backed voles (3) | *B. microti* |  | 3 |  |  |
|  | Hokkaido red-backed vole (1) | *B. microti* |  | 1 |  |  |
|  | Northern red-backed voles (3) | *B. microti* (1), *B. microti-like* (2) | 1 | 3 |  |  |
|  | Japanese grass voles (1) | *B. microti* |  | 1 |  |  |
|  | Smith’s voles (1) | *B. microti-like* |  | 1 |  |  |
|  | Mongolian jirds (1) | *B. microti* |  | 1 |  |  |
| Sorex (2) | Long-clawed shrews (2) | *B. microti* (1), *B. microti-like* (1) | 1 | 2 |  |  |
|  | Dsinezumi shrew (1) | *B. microti-like* | 1 | 1 |  |  |
| Cervids (2) | Sika deer (2) | *B. divergens* (1), *B. divergens-like* (1) |  | 2 |  |  |
| Monkeys (1) | Japanese macaque (1) | *B. microti-like* |  |  |  | 1 |
| Other (1) | Racoons^a^ (1) | *B. microti-like, B.divergens-like* |  | 1 |  |  |
| **China (8)** | | | | | | |
| Rodents (7) | Lesser rice-field rats (1) | *B. microti* |  | 1 |  |  |
|  | White-bellied rats (4) | *B. microti* (4) | 2 | 4 |  |  |
|  | Bower’s white-toothed rats (1) | *B. microti* |  | 1 |  |  |
|  | Brown rats (2) | *B. microti* (1), *B. microti-like* (1) |  | 2 |  |  |
|  | Black rats (3) | *B. microti* (2), *B. microti-like* (1) |  | 3 |  |  |
|  | Tanezumi rats (2) | *B. microti* |  | 2 |  |  |
|  | Yunnan bush rats (1) | *B. microti* |  | 1 |  |  |
|  | Chevrier's field mouse (1) | *B. microti* |  | 1 |  |  |
|  | Ryukyu mice (1) | *B. microti* |  | 1 |  |  |
|  | Sichuan field mice (1) | *B. microti* |  | 1 |  |  |
|  | Striped field mice (2) | *B. microti* | 1 | 2 |  |  |
|  | South China field mice (2) | *B. microti* |  | 2 |  |  |
|  | Eurasian harvest mice (1) | *B. microti* |  | 1 |  |  |
|  | Gairdner’s shrewmouse (2) | *B. microti* |  | 2 |  |  |
|  | Ground squirrels (1) | *B. microti* |  | 1 |  |  |
|  | Dwarf hamsters (1) | *B. microti* |  | 1 |  |  |
|  | Eversmann’s hamsters (1) | *B. microti* |  | 1 |  |  |
|  | European hamsters (1) | *B. microti* |  | 1 |  |  |
|  | Blyth’s voles (1) | *B. microti* |  | 1 |  |  |
|  | Common voles (1) | *B. microti* |  | 1 |  |  |
|  | Oriental voles (2) | *B. microti* |  | 2 |  |  |
|  | Grey red-backed voles (2) | *B. microti* |  | 2 |  |  |
|  | Northern red-backed voles (1) | *B. microti* |  | 1 |  |  |
|  | Yunnan red-backed vole (1) | *B. microti* |  | 1 |  |  |
|  | Southwest China voles (1) | *B. microti* |  | 1 |  |  |
|  | Midday Jirds (1) | *B. microti* |  | 1 |  |  |
|  | Tamarisk Jirds (1) | *B. microti* |  | 1 |  |  |
|  | Ural field mice (1) | *B. microti* |  | 1 |  |  |
|  | Korean field mice (1) | *B. microti* |  | 1 |  |  |
|  | House mice (1) | *B. microti* |  | 1 |  |  |
|  | Mongolian five-toed jerboa (1) | *B. microti* |  | 1 |  |  |
|  | Northern three-toed jerboa (1) | *B. microti* |  | 1 |  |  |
|  | Long-eared jerboa (1) | *B. microti* |  | 1 |  |  |
|  | Yellow lemming (1) | *B. microti* |  | 1 |  |  |
| Sorex (3) | Horsfield’s shrew (1) | *B. microti* |  | 1 |  |  |
|  | Chinese mole shrew (1) | *B. microti* |  | 1 |  |  |
|  | Greater white-toothed shrew (1) | *B. microti* |  | 1 |  |  |
|  | Asian house shrew (2) | *B. microti* |  | 2 |  |  |
|  | Asian gray shrew (1) | *B. microti* |  | 1 |  |  |
|  | Southeast Asian shrew (1) | *B. microti* |  | 1 |  |  |
|  | Ussuri white-toothed shrew (1) | *B. microti* |  | 1 |  |  |
|  | Northern treeshrew (1) | *B. microti* |  | 1 |  |  |
|  | Shrew gymnure (1) | *B. microti* |  | 1 |  |  |
| Bovids (1) | Domestic sheep (1) | *B. crassa-like* |  | 1 |  |  |
| Other (1) | Short-tailed gymnure (1) | *B. microti* |  | 1 |  |  |
| **Russia (8)** | | | | | | |
| Rodents (8) | Striped field mice (1) | *B. microti* |  | 1 |  |  |
|  | Northern birch mice (3) | *B. microti* |  | 3 |  |  |
|  | Korean field mice (2) | *B. microti* |  | 2 |  |  |
|  | Ural field mice (1) | *B. microti* |  | 1 |  |  |
|  | Northern red-backed voles (6) | *B. microti* |  | 6 |  |  |
|  | Bank voles (6) | *B. microti* |  | 6 |  |  |
|  | Grey red-backed voles (7) | *B. microti* |  | 7 |  |  |
|  | Field voles (2) | *B. microti* |  | 2 |  |  |
|  | Tundra voles (2) | *B. microti* |  | 2 |  |  |
|  | Voles^a^ (2) | *B. microti* |  | 2 |  |  |
|  | Siberian chipmunks (1) | *B. microti* |  | 1 |  |  |
|  | Wood lemming (1) | *B. microti* |  | 1 |  |  |
| Sorex (4) | Common shrew (4) | *B. microti* |  | 4 |  |  |
|  | Masked shrew (1) | *B. microti* |  | 1 |  |  |
|  | Eventoothed shrew (1) | *B. microti* |  | 1 |  |  |
|  | Tundra shrew (1) | *B. microti* |  | 1 |  |  |
|  | Eurasian water shrew (1) | *B. microti* |  | 1 |  |  |
|  | Shrews^a^ (2) | *B. microti* |  | 2 |  |  |
| **Republic of Korea (3)** | | | | | | |
| Rodents (3) | Striped field mice (2) | *B. microti* |  | 2 |  |  |
|  | Korean field mice (1) | *B. microti* |  | 1 |  |  |
|  | Grey red-backed vole (1) | *B. microti* |  | 1 |  |  |
|  | Red-cheeked ground squirrels (1) | *B. microti* |  | 1 |  |  |
|  | Yellow lemming (1) | *B. microti* |  | 1 |  |  |
| Cervids (1) | Water deer (1) | *B. microti* |  | 1 |  |  |
| Other (1) | European badgers (1) | *B. microti* |  | 1 |  |  |
| **Iran (1)** | | | | | | |
| Rodents (1) | House mice (1) | *B. microti* |  | 1 |  |  |
| **Iraq (1)** | | | | | | |
| Bovids (1) | Cattle^a^ (1) | *B. divergens* |  | 1 |  |  |
| **Turkey (1)** | | | | | | |
| Bovids (1) | Cattle^a^ (1) | *B. divergens* |  | 1 |  |  |
| **Camobia/Laos/Thailand (1)** | | | | | | |
| Rodents (1) | Ryukyu mice (1) | *B. microti* |  | 1 |  |  |
|  | Fawn-colored mice (1) | *B. microti* |  | 1 |  |  |
|  | Cook’s mice (1) | *B. microti* |  | 1 |  |  |
|  | Greater bandicoot rats (1) | *B. microti* |  | 1 |  |  |
|  | Savile’s bandicoot rats (1) | *B. microti* |  | 1 |  |  |
|  | Bower’s white-toothed rats (1) | *B. microti* |  | 1 |  |  |
|  | Red spiny rats (1) | *B. microti* |  | 1 |  |  |
|  | Chestnut white-bellied rats (1) | *B. microti* |  | 1 |  |  |
|  | Sikkim rats (1) | *B. microti* |  | 1 |  |  |
|  | Ricefield rats (1) | *B. microti* |  | 1 |  |  |
|  | Polynesian rats (1) | *B. microti* |  | 1 |  |  |
|  | Himalayan field rats (1) | *B. microti* |  | 1 |  |  |
|  | Brown rats (1) | *B. microti* |  | 1 |  |  |
| Sorex (1) | Asian house shrews (1) | *B. microti* |  | 1 |  |  |
| Other (1) | Short-tailed gymnure (1) | *B. microti* |  | 1 |  |  |
| **Africa** | | | | | | |
| **Egypt (5)** | | | | | | |
| Rodents (5) | Eastern spiny mice (1) | *B. microti* |  | 1 |  |  |
|  | Brown Rat (3) | *B. microti* |  | 3 |  |  |
|  | Black rat (4) | *B. microti* |  | 4 |  |  |
| **Nigeria (1)** | | | | | | |
| Rodents (1) | Black rats (1) | *B. microti* |  | 1 |  |  |
| **Kenya (2)** | | | | | | |
| Monkeys (2) | Wild baboons (1) | *B. microti* |  | 1 |  |  |
|  | Olive baboons (1) | *B. microti* |  | 1 |  |  |
|  | African green monkeys (1) | *B. microti* |  | 1 |  |  |
| **Tunisia (2)** | | | | | | |
| Bovids (2) | Cattle^a^ (2) | *B. divergens* | **1** | 1 |  | 1 |

^a^ Exact species not specified
